# Comparative transcriptomes reveal pro-survival and cytotoxic programs of mucosal-associated invariant T cells upon Bacillus Calmette–Guérin stimulation

**DOI:** 10.3389/fcimb.2023.1134119

**Published:** 2023-04-06

**Authors:** Manju Sharma, Liang Niu, Xiang Zhang, Shouxiong Huang

**Affiliations:** Department of Environmental and Public Health Sciences, College of Medicine, University of Cincinnati, Cincinnati, OH, United States

**Keywords:** transcriptome, MHC-related protein 1 (MR1), Bacillus Calmette-Guérin (BCG), mucosal-associated invariant T (MAIT) cells, *Mycobacterium tuberculosis* (*M. tuberculosis*)

## Abstract

Mucosal-associated invariant T (MAIT) cells are protective against tuberculous and non-tuberculous mycobacterial infections with poorly understood mechanisms. Despite an innate-like nature, MAIT cell responses remain heterogeneous in bacterial infections. To comprehensively characterize MAIT activation programs responding to different bacteria, we stimulated MAIT cells with *E. coli* to compare with Bacillus Calmette-Guérin (BCG), which remains the only licensed vaccine and a feasible tool for investigating anti-mycobacterial immunity in humans. Upon sequencing mRNA from the activated and inactivated CD8^+^ MAIT cells, results demonstrated the altered MAIT cell gene profiles by each bacterium with upregulated expression of activation markers, transcription factors, cytokines, and cytolytic mediators crucial in anti-mycobacterial responses. Compared with *E. coli*, BCG altered more MAIT cell genes to enhance cell survival and cytolysis. Flow cytometry analyses similarly displayed a more upregulated protein expression of B-cell lymphoma 2 and T-box transcription factor Eomesodermin in BCG compared to *E.coli* stimulations. Thus, the transcriptomic program and protein expression of MAIT cells together displayed enhanced pro-survival and cytotoxic programs in response to BCG stimulation, supporting BCG induces cell-mediated effector responses of MAIT cells to fight mycobacterial infections.

## Introduction

An effective T cell response leads to a low lifetime risk of developing active tuberculosis and a successful vaccine mostly aims to boost protective T cell responses (Cooper, 2009). Conventional T cells are essential for maintaining a low bacterial load, as shown using antibody depletion or adoptive transfer of CD4^+^ or CD8^+^ T cells in mice (Flynn and Chan, 2001; Behar, 2013), increased risk of active tuberculosis with reduced CD4^+^ T cell frequency in HIV co-infection of humans (Cooper, 2009; Prezzemolo et al., 2014), and loss of protection against tuberculosis in primates with CD8^+^ T cell depletion (Chen et al., 2009). However, anti-mycobacterial roles of CD8^+^ T cells in humans remain poorly characterized, and weak peptide-specific CD8^+^ T cell responses have been shown in multiple human vaccine trials (Tameris et al., 2013). Recent findings demonstrated that a large portion of mycobacterial-reactive CD8^+^ T cells in humans are not conventional T cells but mucosal-associated invariant T (MAIT) cells (Gold et al., 2010; Le Bourhis et al., 2010). Unlike conventional T cells that are activated by the peptide antigens presented by major histocompatibility complex (MHC) or human leukocyte antigens (HLA), MAIT cells are activated by non-peptidic small metabolite antigens presented by MHC class I-related protein 1 (MR1) (Huang et al., 2005; Hansen et al., 2007; Huang et al., 2008; Huang et al., 2009; Gold et al., 2010; Le Bourhis et al., 2010; Chua et al., 2011; Young et al., 2013; Huang, 2016; Huang and Moody, 2016; Sharma et al., 2020). Different from the highly variable MHC or HLA proteins for the presentation of eminently divergent peptide antigens, MR1 is monomorphic in humans for the presentation of conserved metabolite antigens such as bacterial riboflavin metabolites, defining the innate-like nature of MAIT cell responses in a donor-unrestricted manner. Stimulation with mycobacterial-infected antigen-presenting cells can strongly enhance the expression of proinflammatory cytokines, surface activation markers, and cytolytic molecules of MAIT cells (Gold et al., 2010; Le Bourhis et al., 2010; Sharma et al., 2020) with poorly known mechanisms, to further bridge the innate and late onset of adaptive immune responses (Meierovics et al., 2013; Huang, 2016; Ioannidis et al., 2020).

MAIT cells are protective against multiple non-tuberculous mycobacterial and *M. tuberculosis* infections (Le Bourhis et al., 2010; Rossjohn et al., 2015; Huang, 2016). Specifically, MAIT cell overexpression in mice inhibits the growth of non-tuberculous *M. abscessus* (Le Bourhis et al., 2010) and *M. bovis* (Chua et al., 2012; Sakala et al., 2015), and partially suppresses *M. tuberculosis* infections (Sakai et al., 2021). In contrast, MR1-knockout mice display a higher load of *M. abscessus* (Le Bourhis et al., 2010), *M. bovis* (Chua et al., 2012; Sakala et al., 2015), and *M. tuberculosis in vivo* (Sakai et al., 2021). These recent findings highlighted MAIT cells as promising targets to induce immune protection against non-tuberculous and tuberculous mycobacterial infections. In the meantime, the *M. bovis* strain Bacillus Calmette-Guerin (BCG) activates MAIT cells rapidly (Chua et al., 2012; Sharma et al., 2020) and is the licensed vaccine against tuberculosis (Raviglione et al., 1995; Oettinger et al., 1999; Li J. et al., 2021). Although BCG vaccination of newborns or toddlers protects children or young adults from pulmonary tuberculosis, its efficacy in higher ages is usually compromised by various factors, including environmental mycobacteria infection (McShane et al., 2012; Kumar, 2021) or an extended period after vaccination (Michelsen et al., 2014; Katelaris et al., 2020). Conventional T cell responses have been a focus for interpreting anti-mycobacterial immunity. BCG vaccination induces antigen-specific CD4^+^ T cell responses critical for regulating cellular and humoral immunity (McShane et al., 2012; Soares et al., 2013). However, CD8^+^ T cell responses to BCG vaccination remain poorly characterized, and peptide-specific CD8^+^ T cells appear at a much smaller scale than CD4^+^ T cells (Murray et al., 2006). This deficit can be likely explained by a high percentage of mycobacterial-specific CD8^+^ T cell clones that have been recently characterized as mucosal-associated invariant T (MAIT) cells (Gold et al., 2010; Le Bourhis et al., 2010), which feature innate-like activation kinetics different from conventional CD8^+^ T cells.

It remains unclear how MAIT cells are stimulated by mycobacteria and develop unique transcriptomic programs to elicit anti-mycobacterial immunity. As known, bacterial activation of MAIT cells is mainly mediated by MR1 presentation of bacterial metabolites (Le Bourhis et al., 2010; Huang, 2016). BCG, *E. coli* (Le Bourhis et al., 2010; Sakala et al., 2015; Sharma et al., 2020), or *Salmonella Typhimurium* (Chen et al., 2017) are expected to provide riboflavin precursor (Kjer-Nielsen et al., 2012; Corbett et al., 2014; Harriff et al., 2018) or other metabolites to be presented by MR1 for MAIT cell activation. Further, MAIT cell activation is depleted by the anti-MR1 antibody blockade of the interaction between the bacterial antigen-loaded MR1 protein and T cell receptor (TCR) (Sharma et al., 2020). The human myelogenous cell line K562 with a defective expression of human leukocyte antigens (HLA) (Lozzio and Lozzio, 1975; Andersson et al., 1979; Roder et al., 1979; Koeffler and Golde, 1980; Li et al., 2019) has been widely used in various studies for testing antigen-specific T cell activation (Escobar et al., 2008; de Jong et al., 2010; de Jong et al., 2014; Sharma et al., 2020; Goodman et al., 2022) and as an ideal antigen-presenting cell for MAIT cell activation upon the overexpression of MR1 protein (Sharma et al., 2020). Similarly, MR1-dependent antigen presentation has been further demonstrated by an impaired anti-bacterial MAIT cell response in MR1 knockout mice (Le Bourhis et al., 2010; Sakala et al., 2015; Chen et al., 2017). Therefore, the co-culture of hMR1-expressing cells with MAIT cells provides a feasible model to investigate human MAIT cell transcriptomes upon the stimulation of MR1-mediated presentation of different bacterial antigens. The comparative MAIT cell transcriptomes stimulated by mycobacteria versus extracellular bacteria are expected to provide MAIT cell activation features and pathways potentially crucial for fighting mycobacterial infections. Recent MAIT cell transcriptomes, including single-cell transcriptomes, have differentiated various MAIT cell subsets in mice (Chandra et al., 2021; Tao et al., 2021) and humans (Vorkas et al., 2022). Moreover, human MAIT cell stimulation through signaling, cytokines, anti-CD3/CD28, or bacterial infections display transcriptomes associated with tissue repair (Hinks et al., 2019; Leng et al., 2019; Chandra et al., 2021; Tao et al., 2021; Vorkas et al., 2022), polyfunctional effector functions (Koay et al., 2019; Lamichhane et al., 2019; Salou et al., 2019; Lee et al., 2020), and innate-like activation programs (Sharma et al., 2020). However, MAIT cells stimulated by MR1 antigen presentation with different bacterial infections or stimulations display heterogeneous responses that have been considered as pathogen selectivity, labeled with diverse sequences of TCRβ chain and an invariant α chain, and attributed to potentially different antigens from various bacteria (Reantragoon et al., 2013; Gold et al., 2014; Jiang et al., 2014; Lepore et al., 2014; Sakala et al., 2015; Meermeier et al., 2016). Differential human MAIT cell response stimulated by BCG vs. *E. coli* is a representative example (Jiang et al., 2014) to further understand the mechanisms contributing to MAIT cell responses to different bacteria. It remains unknown whether mycobacteria stimulate MAIT cell transcriptomes and pathways that are associated with anti-mycobacterial immunity in comparison to extracellular bacteria *E. coli*. Therefore, we profiled the *E. coli*-stimulated MAIT cell transcriptomes to compare with the previously obtained BCG-stimulated MAIT transcriptomes (Sharma et al., 2020). Results demonstrated an enhanced program of pro-survival and cytolytic MAIT cell responses, particularly in BCG stimulation, supporting cell-mediated responses to mycobacterial infection.

## Materials and methods

MAIT cells were activated by bacterial-incubated antigen-presenting cells and sorted for transcriptomic analyses with a summary of analyses provided below and the stepwise procedures detailed in the **Supplementary Materials**. We used human HLA-defective myelogenous leukemia cell line K562 (K562.hMR1) as antigen-presenting cells for bacterial infection and MAIT cell stimulation. Bacterial infection used *Listeria monocytogenes (L. monocytogenes), Escherichia coli (E. coli), Mycobacterium bovis (M. bovis)*, and avirulent *Mycobacterium tuberculosis (M. tuberculosis).* Bacterial-incubated K562.hMR1 cells were co-cultured with anti-Vα7.2-enriched primary human MAIT cells from the blood samples of de-identified healthy donors following the Institutional Review Board (IRB)-approved protocol. The activated MAIT cells were gated on Vα7.2^+^CD161^+^CD4^-^CD8^+^ cells to sort CD69^+^CD26^++^ activated and CD69^+/-^CD26^+/-^ inactivated CD8^+^ MAIT cells for transcriptomic profiling using an Illumina sequencing platform and flow cytometry analyses as we reported (Sharma et al., 2020). In this study, we profiled MAIT cell transcriptomes upon incubating with MAIT-stimulatory *E. coli* and non-stimulatory *Listeria* (accession # pending). The *E. coli*-activated MAIT cell transcriptomes were analyzed in comparison with previously obtained raw RANseq data from BCG-activated MAIT cells (accession # GSE124381), considering the baseline activities of inactivated CD69^+/-^CD26^+/-^ CD8^+^ MAIT cells in the identical and inter-bacterial incubation (Sharma et al., 2020). The obtained transcriptomic data were analyzed using edgeR program for differentially expressed genes (DEG) with calculated p-values, Toppcluster program for gene clustering with Bonferroni correction of p-values, Cytoscape for pathway demonstration, and Gene Set Enrichment Analysis (GSEA) program with enrichment scores and nominal p-values for expression profile comparison. Statistical analyses of flow cytometry results used a pairwise t-test for the directional difference between *E. coli* and BCG stimulations. Detailed materials and methods are described in the **Supplementary Materials**.

## Results

### Differentially expressed genes (DEG) of MAIT cells upon BCG and *E. coli* stimulations

**Figure 1:**
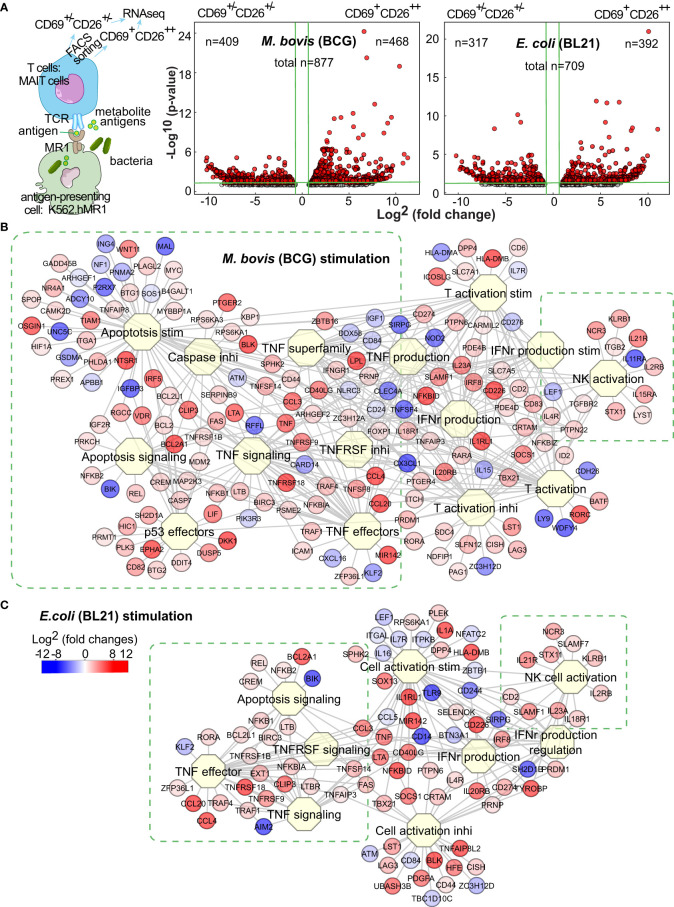
BCG and *E.coli* stimulate different MAIT cell transcriptomes. Anti-Vα7.2-enriched MAIT cells from blood of healthy donors were co-cultured overnight with bacterial-infected K562.hMR1 cells together with anti-CD28 antibody, then gated on Vα7.2^+^CD161^+^CD4^-^CD8^+^ cells, and further sorted into activated versus inactivated MAIT cells labeled with CD69^+^CD26^++^ and CD69^+/-^CD26^+/-^, respectively (**A**, **Figure S1**). The sorted activated vs. inactivated MAIT cells were used for RNA purification and next-generation sequencing. Based on the sequencing results, differentially expressed genes (DEGs) were identified using the edgeR package in R program and shown in Volcano plots as red dots. On the plots, vertical green lines label two-fold changes in intensity counts, and horizontal green lines label p-values of 0.05. For activated MAIT cells between BCG and *E. coli* stimulations, the horizontal green line label a p-value of 0.1 **(A)**. DEGs were used to search biological processes and pathways through the Toppcluster program. Results show multiple functional clusters associated with cell proliferation, apoptosis, cytokine regulation, and cell activation upon BCG and *E. coli* stimulations **(B, C)**.

As various pathogens provide different immune stimuli (Kauffman et al., 2018; Sakai et al., 2021), the determination of differential MAIT transcriptomes stimulated by BCG versus *E. coli* facilitates characterizing anti-mycobacterial immunity of MAIT cells. We have shown that BCG and *E. coli* activated MAIT cells from human blood (Sharma et al., 2020), providing an *in vitro* MAIT cell activation model for intracellular vs. extracellular bacterial stimulation. In this model, human myelogenous leukemia cell line K562 with hMR1 overexpression (K562.hMR1) was incubated with BCG or *E. coli* overnight, washed, and co-cultured with MAIT cells, which were pre-enriched from the peripheral blood of healthy donors using magnetic bead-conjugated anti-Vα7.2 antibody. Upon overnight stimulation with bacterial-incubated K562.hMR1, CD8^+^ MAIT cells as the major peripheral MAIT cell population in humans were gated on Vα7.2^+^CD161^+^CD4^-^CD8^+^ (**Figure S1A**) to sort the activated (CD69^+^CD26^++^) and inactivated (CD69^+/-^CD26^+/-^) MAIT cell subsets using flow cytometry (**Figures 1A**, **S1B**) (Gold et al., 2010; Sharma et al., 2020) for total RNA extraction and mRNA profiling. Identification and separation of activated MAIT cells are based on the expression of CD69 and CD26 upregulated upon the incubation of stimulatory bacteria, but not non-stimulatory *Listeria* or bacterial-free condition (Sharma et al., 2020) (**Figure S1B**). It has been noted that *E. coli* and BCG activate MAIT cells through MR1 presentation of bacterial metabolite antigens to interact with MAIT cell TCR (Le Bourhis et al., 2010; Sakala et al., 2015; Chen et al., 2017; Sharma et al., 2020). Recent studies have also identified riboflavin precursor metabolites from *E. coli, Salmonella*, and *Mycobacterium smegmatis* for MAIT cell activation (Kjer-Nielsen et al., 2012; Corbett et al., 2014; Harriff et al., 2018), but not from non-stimulatory bacteria *Listeria* because of a defective expression of riboflavin synthesis enzymes in *Listeria* (Le Bourhis et al., 2010; Kjer-Nielsen et al., 2012; Gutierrez-Preciado et al., 2015). Further, the upregulation of CD69^+^CD26^++^ on activated MAIT cells upon bacterial stimulation can be largely blocked by anti-MR1 antibody, supporting the dependence of MR1-mediated bacterial antigen presentation for MAIT cell activation (Sharma et al., 2020). Therefore, the validated combinatory marker CD69^+^CD26^++^ was used to label the activated primary human MAIT cells stimulated by MR1-dependent presentation of bacterial antigens (Sharma et al., 2020). Upon RNAseq profiling, gene identities were assigned by aligning the detected sequences with the human genome in GenBank database. We used a robust algorithm of edgeR package in R for multi-factorial comparisons (McCarthy et al., 2012) to generate differentially expressed genes (DEGs) from inactivated MAIT cell subsets and from activated vs. inactivated MAIT cell subsets.

To define a baseline comparator for activated MAIT cells, we generated DEGs for the inactivated MAIT cells with a CD69^+/-^CD26^+/-^ phenotype upon the incubation of MAIT-stimulatory BCG or *E. coli* vs. the non-stimulatory *Listeria* (**Figure S1C**). Both volcano plots suggested the heterogeneity of baseline reactivity with a large number of DEGs between *Listeria* and BCG or *E. coli* incubations (**Figure S1C**) and the potential impact of MR1- or TCR-independent factors from different bacteria, such as pathogen-associated molecular patterns (PAMP). These bacterial factors independent of antigen presentation likely contributed to high numbers of DEGs with inter-bacterial comparison for activated vs. inactivated MAIT cells (**Figure S1D**). Further examining DEGs in inactivated MAIT cells (**Figure S1C**), we found many genes for regulating downstream reactivities, including effector molecules, cytokine receptors, signaling molecules, and exhaustion markers, remained unaltered or minimally altered, although some genes but not proteins for encoding surface markers (e.g., CD69, CD161) were higher in the inactivated MAIT cells from *Listeria* vs. BCG incubation. This heterogeneity of baseline inactivated MAIT cells from *Listeria* incubation is likely due to a broader CD26 expression similar to that in bacterial-free condition (**Figure S1B**), and likely comparable with a portion of pre-activated MAIT cells potentially induced by commensal bacteria in uninfected mice or healthy humans, unlike naïve conventional T cells (Kawachi et al., 2006; Gold et al., 2010; Le Bourhis et al., 2010). Moreover, the upregulated genes from the inactivated MAIT cells upon *Listeria* vs. BCG incubation were analyzed using Enrichr epigenetics enrichment tools (https://maayanlab.cloud/Enrichr/) (Kuleshov et al., 2016) and enriched in the top hit gene sets from thymus tissues or T cells with DNA methylation or histone modification, supporting potential epigenetic regulation in *Listeria* incubation. Thus, inactivated MAIT cells with different bacterial incubation remain heterogeneous. In contrast, MAIT cell subsets from an identically treated condition yielded more homogeneous inactivated MAIT cells to allow accurate analysis and association of altered genes with MR1-dependent MAIT cell activation (**Figure 1A**), because these activated vs. inactivated MAIT subsets responded to the same bacterium and stimulation, underwent identical incubation, staining, and FACS sorting process, and minimized the technical variation. Thus, we focused on the comparative transcriptomes of activated vs. inactivated MAIT cells from the identically treated samples to defined DEGs with a four-fold difference of intensity counts (**Figure 1A**). Several hundred upregulated genes from activated MAIT cells upon BCG and *E. coli* stimulations were shown with volcano plots (**Figure 1A**), in comparison with the inactivated MAIT cells identically stimulated and processed.

### Different gene clusters of MAIT cells upon BCG and *E. coli* stimulations

To determine the gene clusters differentially stimulated by BCG vs. *E. coli*, we used Toppcluster (https://toppcluster.cchmc.org/) (Eisen et al., 1998; Chen et al., 2007) to search DEGs associated with various biological processes and pathways. Although multiple pathways in cytokine production and cell activation are similar (**Figure S1E**), gene clusters involving cell survival, death, and cytolysis differ between BCG and *E. coli* stimulations (**Figure S1E**, **Figures 1B,C**). Overall, BCG stimulation altered more gene clusters in regulating apoptosis and cell life in comparison to *E. coli* stimulation (**Figure S1E**, **Figures 1B,C**), such as tumor necrosis factor α (TNFα) stimulation and signaling, caspase (CASP)-induced apoptosis, and anti-proliferative effects of p53 (**Figure 1B**). These gene clusters include the intrinsic anti-apoptotic genes represented by upregulated B-cell lymphoma 2 (*BCL2*) and the counteracting molecules such as downregulated Bcl-2 interacting killer gene (*BIK)* but upregulated *BLK* (Hegde et al., 1998). *E. coli* altered less number of genes in the clusters regarding cell proliferation or apoptosis (**Figure 1C**). As MAIT cells are cytotoxic T cells, both bacteria upregulated the expression of *GNLY* (granulysin) and *PRF1* (perforin) (Leeansyah et al., 2015) to induce the cytolytic effect of bacterial-infected cells (**Figures 1B,C**). Activated MAIT and natural killer NK cells also co-expressed multiple genes, including *KLRB1* (CD161, Killer cell lectin-like receptor subfamily B, member 1) (Lanier et al., 1994) as an MAIT cell marker (Ussher et al., 2014), *NCR3 (NKp30*) a natural cytotoxicity triggering receptor to interact with CD3ξ (*CD247*) for NK cell differentiation (Siewiera et al., 2015), *SLAMF1* (Signaling lymphocytic activation molecule 1, CD150) as an activation marker (Sharma et al., 2015), ID2 (DNA-binding protein inhibitor ID-2) a transcriptional regulatory protein constitutively expressed in NK cells (Li ZY. et al., 2021), together with genes *LYST* (lysosomal trafficking regulator) and *STX11* (Syntaxin-11) regulating endocytic functions (Valdez et al., 1999; Gil-Krzewska et al., 2016). These data support the overall enhanced cell survival and cytotoxicity of MAIT cells upon BCG and *E. coli* stimulations, respectively. It was reasonable to expect that a direct comparison of the activated MAIT cells between BCG and *E. coli* stimulation generated less degree of difference in gene intensity counts and less altered gene clusters associated with cell reactivity (**Figure S1F**), although thresholds were set more aggressively to potentially visualize this critical difference. However, specific pathway analyses are needed to further understand gene interaction for regulating MAIT cell survival, death, and effector response.

### Enriched pro-survival pathways of MAIT cells upon BCG and *E. coli* stimulations

**Figure 2:**
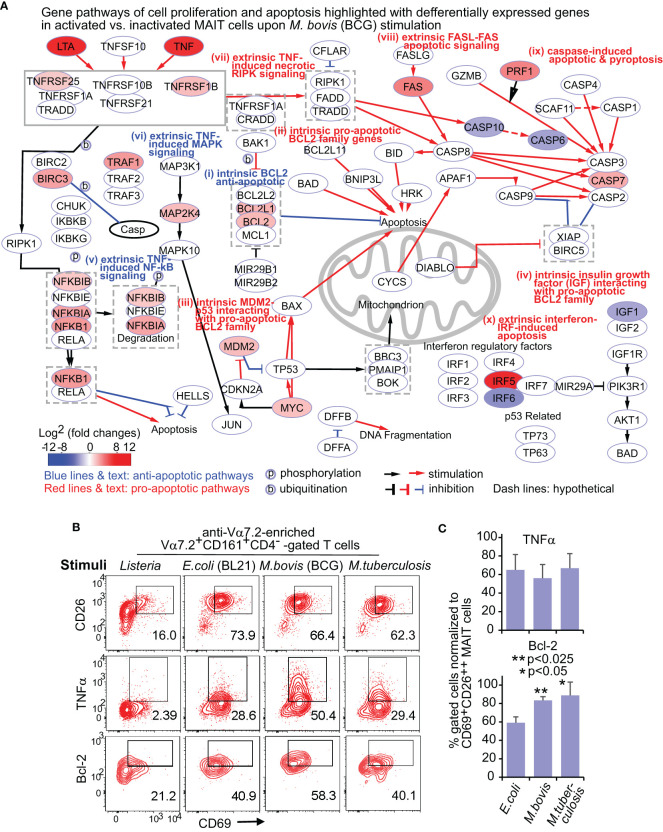
BCG stimulate differential gene and protein expression of MAIT cells in proliferation and apoptosis pathways. DEGs between activated (CD69^+^CD26^++^) subsets versus inactivated (CD69^+/-^CD26^+/-^) subsets of CD8^+^ MAIT cells upon BCG stimulation were annotated in pathways of cell proliferation and apoptosis **(A)**. Protein expression of key cytokine TNFα and key regulatory protein Bcl-2 were confirmed with the percentage of CD69^+^TNFα^+^, and CD69^+^Bcl-2^+^ subsets in comparison to the CD69^+^CD26^++^ subset of CD8^+^ MAIT cells detected by flow cytometry **(B)**. The gating strategy is shown in **Figure S1**. The percentage of CD69^+^TNFα^+^ or CD69^+^Bcl-2^+^ subsets of CD8^+^ MAIT cells was normalized with the percentage of activated CD69^+^CD26^++^ subsets of CD8^+^ MAIT cells. Plots show the means of these normalized percentages and standard errors from multiple donors. A pairwise t-test was used to determine the significance of differences between BCG and *E. coli* stimulations **(C)**.

We used the Cytoscape program to search the network that comprehensively depicts various recently reported pathway mechanisms (labeled by Roman numerals) for regulating cell survival and death (**Figures 2A**; **S2**). The intrinsic pathways include (i) anti-apoptotic *BCL2* family gene members (Czabotar et al., 2014), (ii) pro-apoptotic *BCL2* family gene members (Marsden and Strasser, 2003), and other intrinsic factors acting through *BCL2* family genes for cell growth regulation, such as (iii) MDM2 (mouse double minute 2)-p53 (tumor suppressor gene) and pro-oncogene MYC-p53 counteraction (Wu and Prives, 2018) and (iv) insulin-like growth factor 1 (IGF1) signaling. The extrinsic pathways include TNF signals to mediate (v) proliferative nuclear factor-kappa B (NF-kB), (vi) proliferative mitogen-activated protein kinase (MAPK) pathways (Webster and Vucic, 2020), and (vii) necrotic receptor-interacting protein kinase (RIPK) signaling (Petersen et al., 2015), (viii) FAS-mediated apoptosis (Yamada et al., 2017), (ix) caspase-mediated apoptosis and pyroptosis (Wallach et al., 2014), and (x) interferon-regulatory factor 5 (IRF5)-promoted apoptosis associated with type I interferons (Hu and Barnes, 2009).

DEGs between activated versus inactivated CD8^+^ MAIT cells in BCG stimulation are expected to regulate cell activation, growth or death, cytokines, and cytotoxicity (**Figure 2A**). In the anti-apoptotic *BCL2* gene family (i), the expression of *BCL2*, *BCL2L1 (BCL-X_L_)*, *BCL2A1* (*BFL1*), and *BLK* was enhanced. Favoring cell survival, the expression of pro-apoptotic *BCL2* gene family (ii) was unaltered, including *BID, BIM (BCL2L11), BAD*, and pro-apoptotic “effectors” *BAX* and *BAK1* (Marsden and Strasser, 2003). In MDM2-p53 and MYC-p53 counteractions (iii), enhanced *MDM2* and *MYC* expression could inhibit the pro-apoptotic effect of p53 and stimulate cell growth (Eischen et al., 1999). Further, the reduced expression of insulin-like growth factor 1 (*IGF1*) gene (iv) was associated with a lower activity of the pro-apoptotic protein BAD, further supporting an anti-apoptotic effect of BCG stimulation. Together, enhanced expression of anti-apoptotic genes and unaltered or reduced expression of pro-apoptotic genes in various *BCL2* gene family members and interacting pathways supported a pro-survival effect of MAIT cells in BCG stimulation (**Figure 2A**).

In extrinsic pathways altered by BCG stimulation (**Figure 2A**), the enhanced TNF (TNFα) and lymphotoxin (LTA) cytokines function as both effector molecules in anti-bacterial responses and self-feedback stimuli for regulating cell growth and death. TNF-induced proliferative nuclear factor κB (NF-κB) (v) and mitogen-activated protein kinase (MAPK) (vi) pathways supported a pro-survival effect. BCG induced a lower expression of caspase gene expression (*CASP10* and *CASP6*) following TNF and necrotic RIPK1 signaling (vii), also supporting a pro-survival effect. However, a higher expression of the FAS gene (viii) involved in the self-recognition of FAS ligand could lead to FAS-mediated apoptosis (Yamada et al., 2017). Further, enhanced caspase 7 (*CASP7*) and upstream perforin 1 (*PRF1*) expression involved activation-mediated apoptosis and pyroptosis (Wallach et al., 2014). Enhanced expression of *IRF5* (x) could promote the apoptosis associated with type I interferons (Hu and Barnes, 2009). In addition, the expression of TNF receptor 2 (TNFR2) also named TNF receptor superfamily 1B (*TNFRSF1B*), TNF receptor-associated factor 1 (*TRAF1*), and baculoviral IAP repeat containing 3 (*BIRC3* or cIAP2) were enhanced to play an anti-apoptotic role (Silke and Brink, 2010). Overall, enhanced gene expression was associated with the extrinsic pathways promoting cell survival, with a balance of pro-apoptotic signals for self-activated control of cell growth and death in BCG stimulation (**Figure 2A**).

Although *E. coli* changed the expression of many above genes also altered in BCG stimulation, the expression of anti-apoptotic genes *BCL2L1 (BCL-X_L_), TNFRSF25 (APO3 or DR3), MAP2K4, MDM2, IRF5, IRF6, IGF1*, and the pro-apoptotic gene *CASP7* were unaltered, while the MDM2 suppressor *(CDKN2A)* and FAS ligand *(FASLG)* were upregulated (**Figure S2A**). This difference suggested a more apoptotic effect with *E. coli* than BCG stimulation. We further directly compared the gene expression of the activated CD8^+^ MAIT cell subset upon BCG vs. *E. coli* stimulation. The higher expression of intrinsic *BIM (BCL2L11)* and *CASP4* genes in BCG stimulation compared with *E. coli* stimulation likely suggest a higher baseline gene expression in BCG stimulation for regulating cell survival and apoptosis (**Figure S2B**). Results also showed a higher expression of *BCL2* and *BIRC3* genes, but a lower expression of *CDKN2A* (cyclin-dependent kinase inhibitor 2A) and *IRF6* genes in BCG stimulation, supporting an overall pro-survival transcriptomic program.

### Flow cytometry detected a more enhanced Bcl-2 expression upon BCG vs. *E. coli* stimulation

To confirm the protein expression of key upregulated genes for regulating cell growth or death, we applied flow cytometry to test the expression of TNFα and Bcl-2 proteins in *E. coli*, BCG, and *M. tuberculosis* stimulations. We similarly gated on the CD8^+^ MAIT cells (Vα7.2^+^CD161^+^CD4^-^CD8^+^ cells) as in **Figure S1** and used the negative control *L. monocytogenes* (**Figure 2B**), which is absent of active riboflavin B metabolic pathway for providing an MAIT cell antigen. Because CD69^+^CD26^++^ was used as a combinatory marker to label the total activated MAIT cells (Sharma et al., 2020), we determined % CD69^+^TNFα^+^ or CD69^+^Bcl-2^+^ cells over CD69^+^CD26^++^ CD8^+^ MAIT cells (**Figure 2C**). This similar % of CD69^+^CD26^++^ CD8^+^ MAIT cells supported highly comparable conditions between BCG vs. E. coli stimulation, allowing further examining relative differences of various gene and protein expression. Results showed a higher percentage of Bcl-2-expressing CD8^+^ MAIT cells in mycobacterial than *E. coli* stimulations, supporting the enhanced Bcl-2 protein expression. *Listeria* incubation did not enhance CD69^+^CD26^++^ CD8^+^ MAIT cells and was not included in statistical analyses. TNF protein expression was more variable among donors and not statistically significant for BCG vs. *E. coli* comparison.

### Enriched cytolytic pathways of MAIT cells upon BCG and *E. coli* stimulation

**Figure 3:**
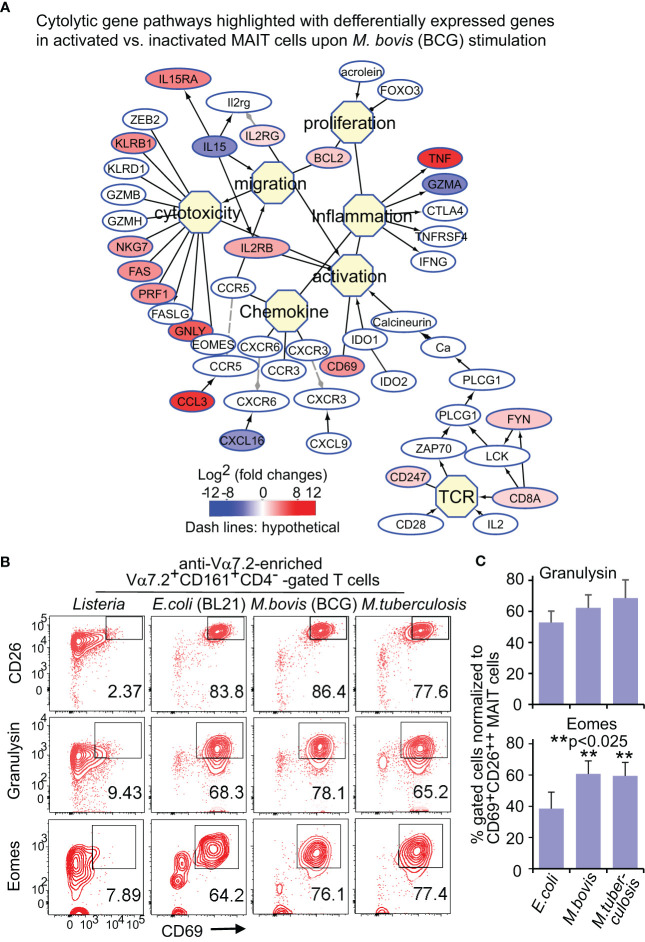
BCG and *E.coli* stimulate differential gene and protein expression of MAIT cells in cytotoxic pathways. Similar to **Figure 2**, DEGs between activated (CD69^+^CD26^++^) subsets versus inactivated (CD69^+/-^CD26^+/-^) subsets of CD8^+^ MAIT cells upon BCG stimulation were annotated in pathways of cytotoxic T cell responses **(A)**. Protein expression of key effect molecule granulysin and key transcription factor Eomes were confirmed with the percentage of CD69^+^granulysin^+^ or CD69^+^Eomes^+^ subsets in comparison to the CD69^+^CD26^++^ subset of CD8^+^ MAIT cells detected by flow cytometry **(B)**. The gating strategy is shown in **Figure S1** as well. The percentage of CD69^+^granulysin^+^ or CD69^+^Eomes^+^ subsets of CD8^+^ MAIT cells were normalized with the percentage of activated CD69^+^CD26^++^ subsets of CD8^+^ MAIT cells. Plots show the means of these normalized percentages and standard error from multiple donors. A pairwise t-test was used to determine the significance of differences between BCG and *E. coli* stimulations **(C)**.

Upon bacterial stimulations, multiple genes involved in the cytolysis of MAIT cells were generally upregulated in BCG stimulation (**Figure 3A**). Beyond the upregulated genes for T cell activation, such as *CD8A* (CD8α), *CD247* (CD3ξ), and *CD69*, cytokine receptor expression was also enhanced, including *IL2RG* (common γ chain)*, IL2RB* (interleukin 2 receptor β subunit), and *IL15RA* (interleukin 15 receptor α subunit). Regarding cytotoxicity, multiple receptors, costimulatory molecules, signaling molecules, and transcription factors were upregulated in bacterial stimulations. CD161, a C-type lectin-like receptor encoded by the upregulated *KLRB1*, labels the maturation and cytotoxicity of NK cells (Lanier et al., 1994; Konjevic et al., 2009; Kurioka et al., 2018). The upregulated *KLRG1* gene encodes a co-inhibitory receptor predominantly on late-differentiated effector and memory CD8^+^ T and NK cells (Nakamura et al., 2009). The enhanced *NKG7* gene encodes natural killer cell granule protein 7, which is a regulator of lymphocyte granule exocytosis and inflammation, such as CD107a (Lamp1 or lysosomal-associated membrane protein-1) translocation to the cell surface for target cell killing (Ng et al., 2020). Multiple genes encoding effector molecules, such as *FAS*, perforin (*PRF1*), and granulysin (*GNLY*), were enhanced differentially in BCG stimulations (**Figure 3A**) for the cytolysis of the infected targeted cells (Lettau and Janssen, 2021). *E. coli* stimulation similarly upregulated cytotoxic genes (**Figure S3A**), with alteration of multiple genes at a lower degree compared with BCG stimulation (**Figure S3B**). Eomesodermin (*EOMES*) was interestingly more upregulated in activated MAIT cells in BCG than *E. coli* stimulation, indicating a higher basal expression of *EOMES* gene in BCG stimulation to promote cytolytic responses.

### Flow cytometry detected a more enhanced Eomes expression upon BCG than *E. coli* stimulation

For protein expression, flow cytometry was used to detect granulysin and Eomes as described in **Figure 2**. Data were similarly analyzed by normalizing % CD69^+^Eomes^+^ CD8^+^ MAIT cells with CD69^+^CD26^++^ CD8^+^ MAIT and showed more enhanced % CD69^+^Eomes^+^ CD8^+^ MAIT cells in BCG stimulation compared with *E. coli*, from one donor (**Figure 3B**) and multiple donors (**Figure 3C**). Together, both gene and protein expression demonstrated upregulated cytolytic molecules to fight intracellular mycobacterial infections.

### MAIT transcriptomes stimulated by BCG are more comparable with conventional CD8^+^ T cells in intracellular microbial infections

**Figure 4:**
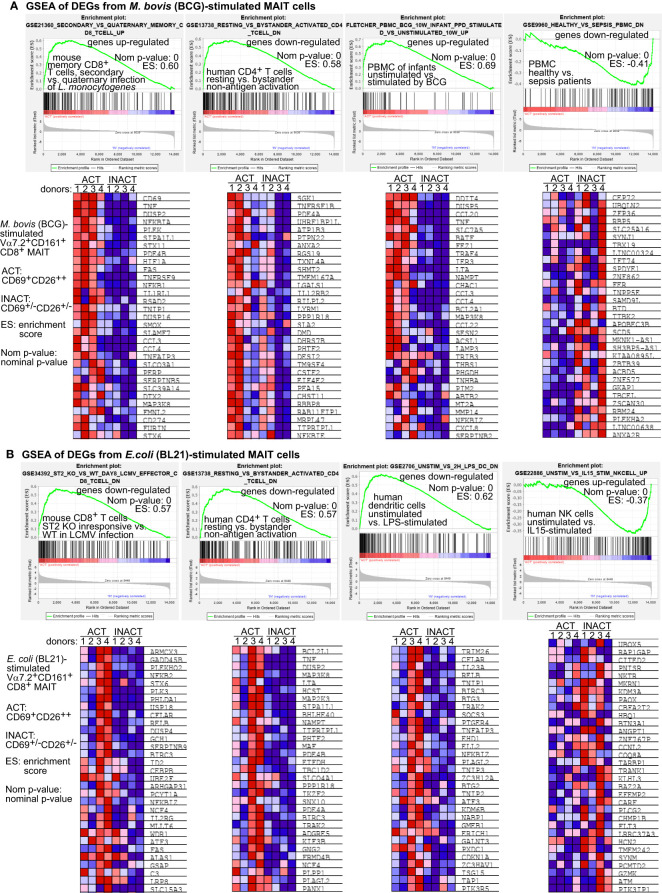
Gene set enrichment analyses (GSEA) support that BCG-stimulated MAIT cell transcriptomes are comparable with CD8^+^ T cells in intracellular microbial infections. BCG-stimulated DEG genes of MAIT cells were analyzed using the GSEA program to identify comparable gene sets with similarly enriched genes from different cell types under various stimulation conditions. The representative gene sets with T cells or other immune cells were selected from the top 20 out of over 4000 enriched gene sets. The representative core-enriched MAIT cell genes shown in heatmaps were selected based on the top running enrichment scores (ES) of the enriched genes **(A)**. *E. coli*-stimulated DEG genes of MAIT cells were similarly analyzed using the GSEA program and shown with representative enrichment plots and enriched genes **(B)**.

To comprehensively compare BCG-stimulated MAIT transcriptomes with other cellular transcriptomes under bacterial or other stimulatory conditions, we performed gene set enrichment analyses (GSEA) for MAIT cell transcriptomes against MSigDB gene expression databases (http://software.broadinstitute.org/gsea/msigdb). The enriched gene sets were cut off with a significant nominal p-value (<0.05) and ranked by the normalized enrichment scores. GSEA analyses generally resulted in around two thousands of such reported gene sets, including various gene sets from conventional CD8^+^ T cells, CD4^+^ T cells, NKT, NK cells, macrophages, dendritic cells, and many other cell types under different stimulation conditions from different biological hosts. Enrichment plots displayed three and one representative gene sets that were selected out of the top twenty ranked gene sets enriched with activated or inactivated MAIT phenotypes, respectively (**Figure 4**). Heatmaps displayed the representative MAIT cell genes from both activated and inactivated MAIT subsets and from the Rank-Ordered List based on the top running enrichment scores (ES) of the tested genes in the targeted gene sets (**Figure 4**). As a result, gene enrichment of MAIT cell transcriptomes in BCG stimulation demonstrated a similar upregulation in activated conventional memory CD8^+^ T cells with intracellular bacterial infection and in PBMCs from infants at ten weeks after BCG vaccination at birth, or a similar downregulation in PBMCs of patients with sepsis, but reversely altered in bystander activated CD4^+^ T cells independent of antigen-presentation (**Figure 4A**). In contrast, MAIT cell genes in *E. coli* stimulation showed reversely altered in viral peptide-activated CD8^+^ T cells, bystander activated CD4^+^ T cells independent of antigens, lipopolysaccharide-stimulated dendritic cells, and IL15-stimulated NK cells (**Figure 4B**). Overall, MAIT cell gene profiles in BCG stimulation were comparable with memory CD8^+^ T cells or PBMCs responding to intracellular bacterial infections. In contrast, MAIT cell gene profiles in *E.coli* stimulation showed a different or reversed association. Results supported similar gene expression of MAIT and other CD8^+^ T cells in response to intracellular pathogens.

## Discussion

Extracellular growing *E. coli* can secrete intermediate metabolites ribityllumazine and ribityluracil from riboflavin biosynthetic pathways to the culture supernatant (Kjer-Nielsen et al., 2012; Corbett et al., 2014; Harriff et al., 2018). These metabolites function as agonist antigens to be loaded to MR1 protein and presented on antigen-presenting cells for MAIT cell activation (McWilliam et al., 2020). Intracellular bacteria such as BCG and *M. tuberculosis* provide antigens for MAIT cell activation, likely involving endocytic compartments for antigen loading and presentation (Huang et al., 2008; Harriff et al., 2016; Huber et al., 2020; Sharma et al., 2020). Our MAIT gene profiles in BCG stimulation showed similar alterations to memory CD8^+^ T cells or PBMCs with intracellular bacterial infections, whereas MAIT cell gene profiles in *E. coli* stimulation showed reversal association with CD8^+^ T cells in intracellular bacterial infections. Transcriptomic analyses on intracellular mycobacterial infections have been mostly focused on macrophages (Nalpas et al., 2015) or monocytes (Kong et al., 2021) for understanding host-pathogen interaction and blood cells from tuberculosis patients. It remains poorly understood how T cell transcriptomes are altered in BCG vaccination or stimulation in a manner dependent on bacterial antigen presentation. As MAIT cells are protective against mycobacterial infections, including *M. tuberculosis* (Sakai et al., 2021), *M. abscessus* (Le Bourhis et al., 2010), and *M. bovis* (Chua et al., 2012; Sakala et al., 2015), this protection likely attributes to MAIT cell responses against intracellular bacterial growth (Le Bourhis et al., 2010; Chua et al., 2012; Sakala et al., 2015; Sakai et al., 2021). Indeed, MAIT cell transcriptomes stimulated by BCG in this study demonstrated pro-survival and cytolytic programs that crucially contribute to the immunity against mycobacterial infections. In the meantime, results discriminate differential MAIT cell transcriptomes responding to intracellular *M. bovis* BCG strain versus extracellular bacteria *E. coli*, suggesting genetic pathways regulating T cell immunity to fight intracellular bacterial infections.

Activated MAIT cells are expected to display pathogen selectivity and ligand discrimination, which have been recently characterized in MAIT cell responses to different bacteria (Reantragoon et al., 2013; Gold et al., 2014; Lepore et al., 2014; Meermeier et al., 2016). MAIT cells express an invariant T cell receptor α chain (TCRα) to recognize conserved antigens in contrast to conventional T cells (Nikolich-Zugich et al., 2004; Logunova et al., 2020). However, the β chain (TCRβ) expresses variable sequences in responses to different bacteria, such as *E. coli* and *Salmonella Typhimurium* that produce typical MAIT cell agonist antigens ribityllumazine and ribityluracil metabolites (Kjer-Nielsen et al., 2012; Corbett et al., 2014; Harriff et al., 2018), versus *Streptococcus pyogenes* that likely generate other unknown MAIT cell antigens (Meermeier et al., 2016). Our chemical purification of bacterial metabolites from BCG also supported the presence of alternative agonists different from the agonists derived from *E. coli* (Corbett et al., 2014). We adapted the HLA-defective myelogenous cell line K562 (Lozzio and Lozzio, 1975; Andersson et al., 1979; Roder et al., 1979; Koeffler and Golde, 1980; Li et al., 2019), widely used as antigen-presentation cells for various T cell activation (Escobar et al., 2008; de Jong et al., 2010; de Jong et al., 2014; Sharma et al., 2020; Goodman et al., 2022), with human MR1 overexpression to present bacterial metabolite antigens for primary MAIT cell activation. Downstream effects of differential pathogen stimulation and variable TCRβ chains (Reantragoon et al., 2013; Gold et al., 2014; Lepore et al., 2014; Meermeier et al., 2016) are expected to induce different MAIT cell transcriptomes that serve as predictors to link stimuli with effector responses. Comparative MAIT cell transcriptomes in this study depicted multifaceted programs regarding MAIT cell activation, survival, apoptosis, and cytolysis, in addition to various cytokine production in BCG vs. *E.coli* stimulation. More specifically, BCG stimulated a higher MAIT cell expression level of *EOMES* and *BCL2* genes and proteins to mediate cytotoxic, cytokine production, and pro-survival programs (**Figures 1**–**3**), which are essential in fighting intracellular mycobacterial infections (Kim et al., 2015; Sharma et al., 2020).

Various DEGs of MAIT cells involved in cell survival and death pathways are astonishing. Although our clustering analyses suggested a broad alteration of these genes, pathway analyses comprehensively displayed altered gene expression in over ten intrinsic and extrinsic gene pathways for regulating cell survival, apoptosis, necrosis, and pyroptosis. Results demonstrated an anti-apoptotic gene expression of MAIT cells in four intrinsic pathways and a balanced survival vs. apoptotic gene expression in extrinsic pathways upon BCG stimulation. In various intrinsic pathways, anti-apoptotic *BCL2* gene family members were enhanced, and other intrinsic factors interacting with *BCL2* gene family members also showed an expression pattern favoring cell survival, such as upregulated *MDM2* and *MYC* expression for a counteraction of p53 protein in BCG stimulation to facilitate MAIT cell proliferation instead of apoptosis or senescence (Wu and Prives, 2018). Multiple extrinsic pathways appear to balance the pro-apoptotic and anti-apoptotic pathways to play crucial roles in MAIT cell homeostasis. The extrinsic signals, including TNFα, LTα, and FAS ligand gene expression enhanced in BCG or *E. coli* stimulation, are common cytokines mediating anti-bacterial effector responses by interacting with corresponding receptors on other effector cells, such as mycobacterial-infected macrophages. If a bacterial infection is under control, these cytokines likely turn back and display a self-feedback control by interacting with their receptors on MAIT cells, leading to homeostatic control of MAIT cell growth by apoptosis or cell death. As both producers and targets of TNF cytokines, T cells can induce the positive feedback of proliferative responses and negative feedback of T cell apoptosis or regulatory T cell differentiation (Locksley et al., 2001; Mehta et al., 2018). TNF production upon BCG stimulation enhanced MAIT cell proliferative pathways mediated by NF-kB and MAPK signaling, but inhibited the necrotic signaling mediated by RIPK1 (receptor-interacting protein or RIP family of serine/threonine protein kinase 1) with reduced downstream caspase expression. TNF effect could be more complex by stimulating a signaling complex of TNF receptor-associated factors (TRAF) and TNFR2 (encoded by *TNFRSF1B*) proteins. For example, BCG enhanced TRAF1 and cellular inhibitor of apoptosis 2 (*cIAP2* or *BIRC3*) to activate NF-κB signaling and prevent TNF-induced apoptosis (Silke and Brink, 2010). However, self-feedback regulation mediated by FAS in BCG stimulation could induce an apoptotic effect. Cell death signals initiated by an upstream cytotoxic molecule FAS mediate the clearance of bacterial-infected cells such as macrophages through MHC class I-restriction by conventional T cells (Kagi et al., 1994) and MR1 restriction by MAIT cells (Boulouis et al., 2020). FAS protein signals, together with the antigen-stimulated TCR signaling, will induce caspase 8-mediated apoptosis and provide feedback control (Bouillet and O’Reilly, 2009; Yamada et al., 2017). In parallel, perforin upregulation occurs with high expression of Tbet in conventional CD8^+^ T cells as reported (Makedonas et al., 2010) and in MAIT cells as we shown (**Figure 1C**). Cytokines IFN stimulate IRF family members together to fight microbial infections, but high IRF5 expression mediating TRAIL (TNF-related apoptosis-inducing ligand) receptors- or death receptor-induced apoptosis functions as negative feedback control (Hu and Barnes, 2009; Fabie et al., 2018). Moreover, lower IGF1 gene expression in the IGF (insulin-like growth factor) signaling pathway is contrasting with higher IRF5 expression, leading to less apoptotic effect in BCG stimulation (**Figure 2A**). Therefore, extrinsic pathways to maintain homeostasis often cross-regulate anti-microbial responses of MAIT cells in fighting infections.

Cytotoxicity-regulatory genes, including natural killer cell group 7 (*NKG7), GNLY, EOMES, TNF*, and *FAS*, were more enhanced in BCG stimulation than *E. coli*. Granulysin, perforin, and TNF function as effector molecules to mediate the cytolytic process of targeted cells. NKG7 interestingly optimizes the exocytosis of lytic granules for the perforin-dependent but not Fas ligand-mediated cytolytic pathway (Morikawa et al., 2021). Eomes is an important transcription factor critical for the formation of effector and memory CD8^+^ T cells (Kaech and Cui, 2012), by mediating the expression of many essential effector molecules, such as perforin, granzymes, and IFNγ (Intlekofer et al., 2005; Banerjee et al., 2010; Pipkin et al., 2010), promoting memory T cell differentiation by inhibiting apoptosis (Banerjee et al., 2010; Kavazovic et al., 2020), and regulating the exhaustion of highly activated CD8^+^ T cells (Li et al., 2018). Enhanced expression of *TBX21*, *EOMES*, and *IL2RB (CD122)* in MAIT cells in this study reflects the similarity of MAIT cells to memory CD8+ T cells or NK cells regarding cytolytic effector responses (Olson et al., 2013). Moreover, cytolytic responses are controlled by multiple different transcription factors, including ID2, Tbet, and Eomes, such as in BCG stimulation, leading to the differentiation of memory, cytolytic, even exhaustion phenotypes.

In this study, we specifically focused on comparing the activated MAIT cells labeled by high CD69 and CD26 expression (CD69^+^CD26^++^) with inactivated MAIT cells in BCG and *E. coli* stimulations to demonstrate MAIT cell transcriptomes dependent on MR1-mediated antigen presentation. Beyond dissecting the transcriptomes of MAIT cell subsets activated by different bacteria, future studies can include bacterial-free controls to assess the global effect of bacterial stimulation on the overall transcriptome. Technically, transcriptomic pathway analyses of T cell responses serve as a tool to integrate stimuli from different sources and facilitate understanding genetic pathways for developing protective anti-bacterial immunity. Consequently, the comparative transcriptomes of MAIT cells in BCG stimulation provided genetic pathways for regulating pro-survival, memory, cytolysis, and exhaustion to elicit anti-mycobacterial MAIT cell immune responses.

## Data availability statement

The data presented in the study are deposited in the GEO repository of the NIH NCBI website (https://www.ncbi.nlm.nih.gov/geo/). Accession GSE228089 is for MAIT cell transcriptomes at E. coli and Listeria incubation conditions. Accession GSE124381 is for MAIT cell transcriptomes at BCG and anti-CD3 stimulation conditions.

## Author contributions

All authors reviewed the manuscript. MS: perform assays; LN: initial transcriptomic data analyses; XZ: RNA sample preparation and sequencing; SH: study design, data analyses, and manuscript writing. All authors contributed to the article and approved the submitted version.

## Supplementary material

Supplementary Figure 1 Gating of activated versus inactivated CD8^+^ MAIT cells for RNA-seq analyses. CD8^+^ MAIT cells were gated on Vα7.2^+^CD161^+^CD4^-^CD8^+^ as Vα7.2^+^CD161^+^ gating has been used in multiple studies to detect MAIT cells (13, 29-32), especially the bacterial-activated MAIT cells (13, 33, 34) **(A)**. % CD69^+^CD26^++^ CD8^+^ MAIT cells and CD69^+/-^CD26^+/-^ cells are annotated, showing the cell populations sorted for RNA-sequencing and the strategy for gating activated MAIT cells **(B)**. To determine whether the CD69^+/-^CD26^+/-^ MAIT subsets at different bacterial incubation conditions show similar background response, we determined DEGs of CD69^+/-^CD26^+/-^ inactivated MAIT cells between *Listeria* and BCG, or between *Listeria* and *E. coli*, suggesting high heterogeneity **(C)**. High numbers of DEGs also occur with the activated MAIT subset (CD69^+^CD26^++^) upon BCG or *E. coli* incubations and inactivated MAIT subset (CD69^+/-^CD26^+/-^) with *Listeria* incubation **(D)**. DEGs from the activated vs. inactivated MAIT cells responding to identical bacterial stimulations, the BCG and *E. coli* stimulation, were more clustered, respectively, to show genes associated with MAIT cell activation and survival **(E)**. DEGs of activated MAIT cells from the direct comparison upon BCG vs. *E. coli* stimulation are expected to have a fewer number of genes and show more narrow clusters associated with MAIT cell reactivities **(F)**.

Supplementary Figure 2 BCG and *E.coli* stimulate differential gene expression of MAIT cells in cell proliferation and apoptosis pathways. Similar to , DEGs between activated (CD69^+^CD26^++^) subsets versus inactivated (CD69^+/-^CD26^+/-^) subsets of CD8^+^ MAIT cells upon *E. coli* stimulation were annotated in pathways of cell proliferation and apoptosis **(A)**. DEGs of activated MAIT cells between BCG and *E.coli* stimulation are also annotated in pathways of cell proliferation and apoptosis **(B)**.

Supplementary Figure 3 BCG and *E.coli* stimulate differential gene expression of MAIT cells in cytotoxic pathways. Similar to , DEGs between activated (CD69^+^CD26^++^) subsets versus inactivated (CD69^+/-^CD26^+/-^) subsets of CD8^+^ MAIT cells upon *E. coli* stimulation were annotated in pathways of cell activation and cytotoxicity **(A)**. DEGs of activated MAIT cells between BCG and *E.coli* stimulation were annotated in pathways of cytotoxic T cell responses **(B)**.
