# Supplementary material for: Comparative transcriptomes reveal pro-survival and cytotoxic programs of mucosal-associated invariant T cells upon Bacillus Calmette–Guérin stimulation
Source: Front Cell Infect Microbiol. 2023 Apr 6;13:1134119. doi: 10.3389/fcimb.2023.1134119 (PMC10116416; doi:10.3389/fcimb.2023.1134119)

Supplementary Material

Comparative transcriptomes reveal pro-survival and cytotoxic programs of mucosal-associated invariant T cells upon Bacillus Calmette–Guérin stimulation

*Manju Sharma, Liang Niu, Xiang Zhang, Shouxiong Huang**

Department of Environmental and Public Health Sciences, University of Cincinnati College of Medicine, Cincinnati, OH 45267

**Immunobiology graduate program, Cincinnati Children's Hospital, Cincinnati, OH 45267*

Correspondence address:

Shouxiong Huang

Department of Environmental Health

University of Cincinnati College of Medicine

251 Kettering Laboratory

160 Panzeca Way

Cincinnati, OH 45267

Email: Shouxiong.huang@uc.edu

Phone: 513-558-7572

Fax: 513-558-4397

Keywords: mucosal-associated invariant T (MAIT) cells; transcriptome; MHC-related protein 1 (MR1); *Mycobacterium bovis*; *E. coli.*

Running title: MAIT transcriptomes in BCG *and E.coli* infections

Supplementary Materials and Methods:

Preparation of MR1-overexpressing cells. K562 is a human HLA-defective (1) myelogenous leukemia cell line with multiple potentials, including an erythroblast potential, for myeloid cell differentiation (2-4). K562 has been a widely used antigen-presenting cell for studying T cell activation, including conventional T cells (5, 6), CD1-restricted T cells (7, 8), and MR1-restricted T cells (9). We have demonstrated that K562 cells with human MR1 overexpression (K562.hMR1) and bacterial incubation activated monoclonal and polyclonal human MAIT cells dependent on the antigen presentation mediated by the human MR1 protein (9). Using this co-culture model, we defined the combinatory surface marker $CD26^{++}CD69^{+}$ for labeling the activated human MAIT cells upon bacterial incubation in a manner dependent on MR1 antigen presentation. More specifically, upon the incubation with *E. coli*, BCG, *M. tuberculosis*, the overexpressed MR1 protein efficiently activates MAIT cells through presenting small bacterial metabolites such as riboflavin precursor or other metabolites (9-12). *Listeria monocytogenes (L. monocytogenes)* was used as a negative control due to its defective expression of riboflavin metabolic enzymes for antigenic metabolite synthesis (10, 13, 14). These bacterial-activated MAIT cells can be shown with an enhanced expression of surface markers CD26 and CD69, production of various cytokines, and the reduced intensity of the infected fluorescence-labeled BCG (9). Taking advantage of the K562.hMR1-induced human MAIT cell activation model dependent on the MR1 presentation of bacterial metabolite antigens and the defined activation marker $CD26^{++}CD69^{+}$ (9), we applied this validated *in vitro* co-culture model to understand the differential MAIT cell transcriptomes upon bacterial stimulation in this study. We overexpressed human MR1 protein in the K562 cell (K562.hMR1) (1) using retroviral transduction as we reported (15). Human MR1 gene was cloned into retroviral vector pMXIP to generate retrovirus by transfecting human embryonic kidney 293T cells (16). The retrovirus was further used to infect K562 cells, which were selected with 10 ug/ml puromycin for the retroviral-infected K562 cells. The expression of recombinant human MR1 was confirmed with flow cytometry using anti-hMR1 antibody (clone 26.5) (9, 15-17). K562.hMR1 was incubated with bacteria *E. coli*, BCG, and *L. monocytogenes*, washed with culture media, and used as an antigen-presenting cell for MAIT cell activation.

Bacterial incubation of K562.hMR1 cells. We incubated K562.hMR1 cells with *Listeria monocytogenes (L. monocytogenes* strain J0161, Bei resources*)*, *Escherichia coli (*non-pathogenic *E. coli* strain BL21, New England BioLabs), *Mycobacterium bovis-* (*M. bovis-*) derived Bacille Calmette-Guerin (BCG) vaccine strain (Pasteur) (18), and avirulent *Mycobacterium tuberculosis* (*M. tuberculosis*, strain H37Ra defective in ESX-1 secretion (19), Colorado State University, Fort Collins, CO) (20). We grew mycobacterial strains BCG and H37Ra for 5 to 6 days using middlebrook 7H9 complete medium in an orbital shaker at 37 ^{o}C and a speed of 270 rpm. *E.coli* and *L. monocytogenes* were cultured overnight using Luria-Bertani broth in an orbital shaker at 37 ^{o}C and a speed of 100 rpm. We harvested bacteria at the log growth phase, washed with phosphate buffer saline (PBS), and measured their absorbance (optical density at wavelength 600 nanometres, OD_{600}) according to the report (21). OD_{600} provides a semi-quantitative measurement of bacterial cell numbers for MAIT cell activation (21). K562.hMR1 cells were incubated with *E.coli* and *Listeria* at an estimated multiplicity of infection (MOI, for cell to bacteria ratio) of 1:10 and with BCG or H37Ra at MOI of 1:100, considering a much faster growing rate of *E. coli* than mycobacteria during the overnight incubation.

Isolation and activation of primary human MAIT cells. We obtained blood samples of healthy donors with written informed consent at the Hoxworth Blood Center in the University of Cincinnati and processed the de-identified blood samples according to the approved protocols by the Institutional Review Board. We then isolated human peripheral blood mononuclear cells (PBMCs) using Ficoll-paque gradient (GE Healthcare), incubated them with anti-Vα7.2 antibody (3C10) conjugated with PE (Biolegend), and performed positive selection with anti-PE antibody-conjugated magnetic beads (MACS, Miltenyi Biotec) according to manufacturer's instructions. These anti-Vα7.2-enriched primary human MAIT cells were co-cultured with bacterial-incubated K562.hMR1 cells in a ratio of 4:1 for around 15 hours together with anti-CD28 (clone CD28.2) antibody at 2ug/ml for co-stimulation. Strong MAIT cell activation in this assay was measured with upregulated $CD69^{+}CD26^{++}$ MAIT cells as we reported (9) using anti-CD3/CD28 activation as a positive control. The dependence on MR1-mediated antigen presentation was controlled with the conditions of anti-MR1 antibody (clone 26.5) blockade (22, 23) and negative control bacterium *L. monocytogenes*, which was previously shown to be unable to activate MAIT cells (9) due to lack of metabolic pathways to produce riboflavin metabolites (10, 24).

RNA-seq of human MAIT cells. Upon co-culture of bacterial-incubated K562.hMR1 and MAIT cells, MAIT cells from three donors were first gated on Vα7.2$^+$CD161$^+$CD4$^-$CD8$^+$ as our targeted major MAIT cell subset in this study and further sorted based on CD69$^+$CD26^{++} and CD69$^-$CD26$^{+/-}$ into activated versus inactivated MAIT cells (Fig. S1). Around one thousand cells were collected for each subset and lysed in the Lysis Buffer for total RNA extraction using mirVana kit (ThermoFisher, Grand Island, NY). Agilent Bioanalyzer and RNA 6000 Pico chip (Agilent, Santa Clara, CA) measured RNA integrity and showed a high quality of samples. NEBNext Poly(A) mRNA Magnetic Isolation Module (New England BioLabs, Ipswich, MA) was used to purify polyA RNA. RNA-seq library was prepared using NEBNext Ultra Directional RNA Library Prep kit (New England BioLabs, Ipswich, MA) with incorporated dUTP during the second cDNA synthesis to maintain strand specificity and with 15 cycles of PCR to enrich and index the library. Upon clean up and quality control analysis with Bioanalyzer for the amplified libraries and negative control, individually indexed and compatible libraries at 15 pM total were proportionally pooled for clustering on single-read flow cells in cBot system (Illumina, San Diego, CA). RNA-seq of the clustered libraries targeted to generate 51 bp reads at ~25 million per sample. RNA-seq data can be accessed at the GEO database (accession number GSE124381 for BCG infection and accession number for *E. coli* infection).

Analyses of differentially expressed genes (DEGs). Similar to our previous report (22), transcriptomic data were analyzed to identify DEGs, which were further used for pathway analyses. To identify DEGs, we aligned the sequence reads to the genome and converted them to intensity counts. EdgeR program on the Bioconductor R platform was used to compare resulted intensity counts to identify DEGs using a robust algorithm in R package, glmFit， a genewise negative binomial generalized linear model, to minimize raw data alteration and allow the comparison of multi-factorial comparisons among multiple conditions (25). We generated DEGs based on intensity fold changes (>2 folds) at a p-value of <0.05 between activated and inactivated CD8$^+$MAIT cells in each bacterial stimulation measured by the surface markers of CD69$^+$CD26^{++} and CD69$^-$CD26$^{+/-}$. We also compare genes with more than 2 folds of intensity changes between BCG and *E. coli* stimulations with CD69$^+$CD26^{++} CD8$^+$MAIT cells based on a p-value of <0.1 to allow the variation from

different batches of cell culture, bacterial stimulation, cell sorting, and gene sequencing. DEGs were shown with volcano plots generated with edgeR and ggplot2 programs on the R platform. Upregulated and downregulated genes between different stimulation conditions were compared using Venn diagrams. To predict shared functional clusters and pathways of DEGs, we applied ToppCluster program based on hypergeometric tests for functional enrichment (https://toppcluster.cchmc.org/) (26) to search DEGs against various databases of gene pathways and obtain multiple clusters of genes associated with different cellular pathways at a statistically significant level of Bonferroni p-value (<0.05). Resulted gene clusters of different cellular pathways constructed with a Fruchterman-Reingold layout algorithm were further visualized with Cytoscape software Version 3.3.0 (www.cytoscape.org/), a broadly used open-source software platform for visualizing complex networks.

Gene pathway and enrichment analyses To analyze the involved pathways for MAIT cell DEGS in BCG vs. *E. coli* stimulations, we applied Cytoscape to search various databases, including PANTHER, MSigDB, KEGG, NCI Pathway, and Reactome databases, to identify optimal and comprehensive pathways for immune cell survival, proliferation, apoptosis, and cytotoxicity. Two hit pathways, the cell growth and death pathway and cytotoxicity pathway, were further verified by a serial relevant publications. DEGs of MAIT cells stimulated in BCG vs. *E. coli* stimulations were annotated onto both pathways by coloring DEGs based on folds of alteration. Gene enrichment analyses can comprehensively determine the programmatic and predict functional similarity based on the directionally altered gene expression in bacterial-stimulated MAIT cells and other cell types with specific stimulations. To perform the enrichment analyses of MAIT DEG genes, we used Gene Set Enrichment Analysis (GSEA) program (https://www.gsea-msigdb.org/gsea/index.jsp) to search various gene sets of different immune and other cell types in human MsigDB database (27). Using GSEA program 4.0.2, we searched the identified BCG- and *E. coli*-induced DEGs against MsigDB gene expression databases (http://software.broadinstitute.org/gsea/msigdb). The enrichment gene sets were exported based on a significant nominal p-value less than 0.05 and ranked based on normalized enrichment score (NES), which accounts for differences in gene set size and in gene correlations between the enriched gene sets and the tested dataset (28). The enriched gene sets with a significant nominal p-value (<0.05) were ranked by the normalized enrichment scores. Enrichment plots were used to show the representative enriched gene sets that

were selected at three out of top twenty ranked gene sets enriched with activated phenotypes and one out of twenty ranked gene sets enriched with inactivated phenotypes. Further, heatmaps were used to show the representative MAIT cell genes from activated vs. inactivated subsets based on the top running enrichment scores (ES) of gene set members from the Rank-Ordered List (Fig. 4).

Antibodies and flow cytometry for human MAIT cells. Surface activation markers, intracellular cytokines, and transcription factors of MAIT cells were shown with fluorescence-labeled antibodies following the manufacturer's instructions (Biolegend unless noted) and detected using flow cytometry. Briefly, Brefeldin A (10ug/ml) was used to inhibit protein transport from the endoplasmic reticulum to the Golgi complexes and added 2 hours prior to cell harvesting. Cells were then harvested and washed twice with staining buffer (PBS with 2% FBS) then blocked with anti-human Fc receptor antibodies, including anti-CD64 (mouse IgG1, clone 10.1), CD32 (mouse IgG2b, clone FUN-2), CD16 (mouse IgG1, clone 3G8), and additional Fc receptor blocking solution human TruStain FcX. We started with surface staining by adding fluorescence-labeled monoclonal antibodies (clone numbers), including phycoerythrin (PE)-Vα7.2, biotin-CD4 (OKT4) and streptavidin-conjugated quantum dot 525 or brilliant violet 510-CD4, brilliant violet 711-CD8α (RPA-T8), Allophycocyanin/Cyanine7 (APC/Cy7)-CD161 (HP-3G10), brilliant violet 605 or 421-CD69 (FN50), and PE/Cy5-CD26 (BA5b), to incubate cells for 30 min at 4°C in dark. To further stain the intracellular cytokines and transcription factors, cells coated with antibodies on the surface were further fixed and permeabilized using the Fix/Perm Kit (Biolegend) and stained with fluorescence-labeled antibodies in the 1 x Perm buffer for 30 minutes at 4°C in dark. These anti-cytokine and anti-transcription factor antibodies include PE/Cy7-TNF-α (MAb11), FITC-Bcl-2 (BCL/10C4), Alexa fluor 647-granulysin (DH2), and Alexa fluor 488-Eomes (644730, R&D systems). Flow cytometry used BD Fortessa and Millipore Guava EasyCyte 12 channel high throughput flow cytometer according to the manufacturer's instructions. Flow cytometry data were further compensated and analyzed using Millipore Guava incyte and FlowJo software programs. Directional differences between pairwise samples of mycobacterial and *E. coli* stimulations from the same donor was statistically tested using a paired t-test.

Supplementary Figure legends:

Fig. S1. Gating of activated versus inactivated CD8^{+} MAIT cells for RNA-seq analyses. CD8^{+} MAIT cells were gated on Vα7.2^{+}CD161^{+}CD4^{-}CD8^{+} as Vα7.2^{+}CD161^{+} gating has been used in multiple studies to detect MAIT cells (13, 29-32), especially the bacterial-activated MAIT cells (13, 33, 34) (A). % CD69^{+}CD26^{++} CD8^{+} MAIT cells and CD69$^{+/-}$CD26$^{+/-}$ cells are annotated, showing the cell populations sorted for RNA-sequencing and the strategy for gating activated MAIT cells (B). To determine whether the CD69$^{+/-}$CD26$^{+/-}$ MAIT subsets at different bacterial incubation conditions show similar background response, we determined DEGs of CD69$^{+/-}$CD26$^{+/-}$ inactivated MAIT cells between *Listeria* and BCG, or between *Listeria* and *E. coli*, suggesting high heterogeneity (C). High numbers of DEGs also occur with the activated MAIT subset (CD69^{+}CD26^{++}) upon BCG or *E. coli* incubations and inactivated MAIT subset (CD69$^{+/-}$CD26$^{+/-}$) with *Listeria* incubation (D). DEGs from the activated vs. inactivated MAIT cells responding to identical bacterial stimulations, the BCG and *E. coli* stimulation, were more clustered, respectively, to show genes associated with MAIT cell activation and survival (E). DEGs of activated MAIT cells from the direct comparison upon BCG vs. *E. coli* stimulation are expected to have a fewer number of genes and show more narrow clusters associated with MAIT cell reactivities (F).

Fig. S2. BCG and *E.coli* stimulate differential gene expression of MAIT cells in cell proliferation and apoptosis pathways. Similar to Fig. 2, DEGs between activated (CD69^{+}CD26^{++}) subsets versus inactivated (CD69$^{+/-}$CD26$^{+/-}$) subsets of CD8^{+} MAIT cells upon *E. coli* stimulation were annotated in pathways of cell proliferation and apoptosis (A). DEGs of activated MAIT cells between BCG and *E.coli* stimulation are also annotated in pathways of cell proliferation and apoptosis (B).

Fig. S3. BCG and *E.coli* stimulate differential gene expression of MAIT cells in cytotoxic pathways. Similar to Fig. 3, DEGs between activated (CD69^{+}CD26^{++}) subsets versus inactivated (CD69$^{+/-}$CD26$^{+/-}$) subsets of CD8^{+} MAIT cells upon *E. coli* stimulation were annotated in pathways of cell activation and cytotoxicity (A). DEGs of activated MAIT cells between BCG and *E.coli* stimulation were annotated in pathways of cytotoxic T cell responses (B).

Supplementary References:

1. Roder JC, Ahrlund-Richter L, and Jondal M. Target-effector interaction in the human and murine natural killer system: specificity and xenogeneic reactivity of the solubilized natural killer-target structure complex and its loss in a somatic cell hybrid. *J Exp Med* (1979) **150**(3)**:** 471-481.
2. Andersson LC, Nilsson K, and Gahmberg CG. K562--a human erythroleukemic cell line. *Int J Cancer* (1979) **23**(2)**:** 143-147. doi:10.1002/ijc.2910230202
3. Koeffler HP, and Golde DW. Human myeloid leukemia cell lines: a review. *Blood* (1980) **56**(3)**:** 344-350.
4. Li G, Bethune MT, Wong S, Joglekar AV, Leonard MT, Wang JK, et al. T cell antigen discovery via trogocytosis. *Nat Methods* (2019) **16**(2)**:** 183-190. doi:10.1038/s41592-018-0305-7
5. Goodman DB, Azimi CS, Kearns K, Talbot A, Garakani K, Garcia J, et al. Pooled screening of CAR T cells identifies diverse immune signaling domains for next-generation immunotherapies. *Sci Transl Med* (2022) **14**(670)**:** eabm1463. doi:10.1126/scitranslmed.abm1463
6. Escobar H, Crockett DK, Reyes-Vargas E, Baena A, Rockwood AL, Jensen PE, et al. Large scale mass spectrometric profiling of peptides eluted from HLA molecules reveals N-terminal-extended peptide motifs. *J Immunol* (2008) **181**(7)**:** 4874-4882. doi:10.4049/jimmunol.181.7.4874
7. de Jong A, Cheng TY, Huang S, Gras S, Birkinshaw RW, Kasmar AG, et al. CD1a-autoreactive T cells recognize natural skin oils that function as headless antigens. *Nat Immunol* (2014) **15**(2)**:** 177-185. doi:10.1038/ni.2790
8. de Jong A, Pena-Cruz V, Cheng TY, Clark RA, Van Rhijn I, and Moody DB. CD1a-autoreactive T cells are a normal component of the human alphabeta T cell repertoire. *Nat Immunol* (2010) **11**(12)**:** 1102-1109. doi:10.1038/ni.1956
9. Sharma M, Zhang S, Niu L, Lewinsohn DM, Zhang X, and Huang S. Mucosal-Associated Invariant T Cells Develop an Innate-Like Transcriptomic Program in Anti-mycobacterial Responses. *Front Immunol* (2020) **11:** 1136. doi:10.3389/fimmu.2020.01136
10. Kjer-Nielsen L, Patel O, Corbett AJ, Le Nours J, Meehan B, Liu L, et al. MR1 presents microbial vitamin B metabolites to MAIT cells. *Nature* (2012). doi:10.1038/nature11605
11. Corbett AJ, Eckle SB, Birkinshaw RW, Liu L, Patel O, Mahony J, et al. T-cell activation by transitory neo-antigens derived from distinct microbial pathways. *Nature* (2014) **509**(7500)**:** 361-365. doi:10.1038/nature13160
12. Harriff MJ, McMurtrey C, Froyd CA, Jin H, Cansler M, Null M, et al. MR1 displays the microbial metabolome driving selective MR1-restricted T cell receptor usage. *Sci Immunol* (2018) **3**(25). doi:10.1126/sciimmunol.aao2556
13. Le Bourhis L, Martin E, Peguillet I, Guihot A, Froux N, Core M, et al. Antimicrobial activity of mucosal-associated invariant T cells. *Nat Immunol* (2010) **11**(8)**:** 701-708. doi:10.1038/ni.1890
14. Gutierrez-Preciado A, Torres AG, Merino E, Bonomi HR, Goldbaum FA, and Garcia-Angulo VA. Extensive Identification of Bacterial Riboflavin Transporters and Their Distribution across Bacterial Species. *PLoS One* (2015) **10**(5)**:** e0126124. doi:10.1371/journal.pone.0126124
15. Huang S, Gilfillan S, Cella M, Miley MJ, Lantz O, Lybarger L, et al. Evidence for MR1 antigen presentation to mucosal-associated invariant T cells. *J Biol Chem* (2005) **280**(22)**:** 21183-21193. doi:10.1074/jbc.M501087200
16. Huang S, Martin E, Kim S, Yu L, Soudais C, Fremont DH, et al. MR1 antigen presentation to mucosal-associated invariant T cells was highly conserved in evolution. *Proc Natl Acad Sci U S A* (2009) **106**(20)**:** 8290-8295. doi:10.1073/pnas.0903196106
17. Huang S, Gilfillan S, Kim S, Thompson B, Wang X, Sant AJ, et al. MR1 uses an endocytic pathway to activate mucosal-associated invariant T cells. *J Exp Med* (2008) **205**(5)**:** 1201-1211. doi:10.1084/jem.20072579
18. Layre E, Lee HJ, Young DC, Martinot AJ, Buter J, Minnaard AJ, et al. Molecular profiling of Mycobacterium tuberculosis identifies tuberculosinyl nucleoside products of the virulence-associated enzyme Rv3378c. *Proc Natl Acad Sci U S A* (2014) **111**(8)**:** 2978-2983. doi:10.1073/pnas.1315883111
19. Conrad WH, Osman MM, Shanahan JK, Chu F, Takaki KK, Cameron J, et al. Mycobacterial ESX-1 secretion system mediates host cell lysis through bacterium contact-dependent gross membrane disruptions. *Proceedings of the National Academy of Sciences* (2017) **114**(6)**:** 1371-1376. doi:doi:10.1073/pnas.1620133114
20. Roura-Mir C, Wang L, Cheng TY, Matsunaga I, Dascher CC, Peng SL, et al. Mycobacterium tuberculosis regulates CD1 antigen presentation pathways through TLR-2. *J Immunol* (2005) **175**(3)**:** 1758-1766.

21. Biesta-Peters EG, Reij MW, Joosten H, Gorris LG, and Zwietering MH. Comparison of two optical-density-based methods and a plate count method for estimation of growth parameters of Bacillus cereus. *Appl Environ Microbiol* (2010) **76**(5)**:** 1399-1405. doi:10.1128/AEM.02336-09
22. Sharma M, Zhang X, Zhang S, Niu L, Ho SM, Chen A, et al. Inhibition of endocytic lipid antigen presentation by common lipophilic environmental pollutants. *Scientific reports* (2017) **7**(1)**:** 2085. doi:10.1038/s41598-017-02229-7
23. Sharma M, Zhang X, and Huang S. Integrate Imaging Flow Cytometry and Transcriptomic Profiling to Evaluate Altered Endocytic CD1d Trafficking. *J Vis Exp* (2018)(140). doi:10.3791/57528
24. Eckle SB, Corbett AJ, Keller AN, Chen Z, Godfrey DI, Liu L, et al. Recognition of Vitamin B Precursors and Byproducts by Mucosal Associated Invariant T Cells. *J Biol Chem* (2015) **290**(51)**:** 30204-30211. doi:10.1074/jbc.R115.685990
25. McCarthy DJ, Chen Y, and Smyth GK. Differential expression analysis of multifactor RNA-Seq experiments with respect to biological variation. *Nucleic Acids Res* (2012) **40**(10)**:** 4288-4297. doi:10.1093/nar/gks042
26. Chen J, Xu H, Aronow BJ, and Jegga AG. Improved human disease candidate gene prioritization using mouse phenotype. *BMC Bioinformatics* (2007) **8:** 392. doi:10.1186/1471-2105-8-392
27. Subramanian A, Tamayo P, Mootha VK, Mukherjee S, Ebert BL, Gillette MA, et al. Gene set enrichment analysis: a knowledge-based approach for interpreting genome-wide expression profiles. *Proc Natl Acad Sci U S A* (2005) **102**(43)**:** 15545-15550. doi:10.1073/pnas.0506580102
28. Cantu E, Lederer DJ, Meyer K, Milewski K, Suzuki Y, Shah RJ, et al. Gene set enrichment analysis identifies key innate immune pathways in primary graft dysfunction after lung transplantation. *Am J Transplant* (2013) **13**(7)**:** 1898-1904. doi:10.1111/ajt.12283
29. Sharma PK, Wong EB, Napier RJ, Bishai WR, Ndung'u T, Kasprowicz VO, et al. High expression of CD26 accurately identifies human bacteria-reactive MR1-restricted MAIT cells. *Immunology* (2015) **145**(3)**:** 443-453. doi:10.1111/imm.12461
30. Leeansyah E, Svard J, Dias J, Buggert M, Nystrom J, Quigley MF, et al. Arming of MAIT Cell Cytolytic Antimicrobial Activity Is Induced by IL-7 and Defective in HIV-1 Infection. *PLoS Pathog* (2015) **11**(8)**:** e1005072. doi:10.1371/journal.ppat.1005072
31. Ben Youssef G, Tourret M, Salou M, Ghazarian L, Houdouin V, Mondot S, et al. Ontogeny of human mucosal-associated invariant T cells and related T cell subsets. *J Exp Med* (2018). doi:10.1084/jem.20171739
32. Dusseaux M, Martin E, Serriari N, Peguillet I, Premel V, Louis D, et al. Human MAIT cells are xenobiotic-resistant, tissue-targeted, CD161hi IL-17-secreting T cells. *Blood* (2011) **117**(4)**:** 1250-1259. doi:10.1182/blood-2010-08-303339
33. Lamichhane R, Schneider M, de la Harpe SM, Harrop TWR, Hannaway RF, Dearden PK, et al. TCR- or Cytokine-Activated CD8(+) Mucosal-Associated Invariant T Cells Are Rapid Polyfunctional Effectors That Can Coordinate Immune Responses. *Cell Rep* (2019) **28**(12)**:** 3061-+. doi:10.1016/j.celrep.2019.08.054
34. Gold MC, Cerri S, Smyk-Pearson S, Cansler ME, Vogt TM, Delepine J, et al. Human mucosal associated invariant T cells detect bacterially infected cells. *PLoS Biol* (2010) **8**(6)**:** e1000407. doi:10.1371/journal.pbio.1000407

Fig. S1

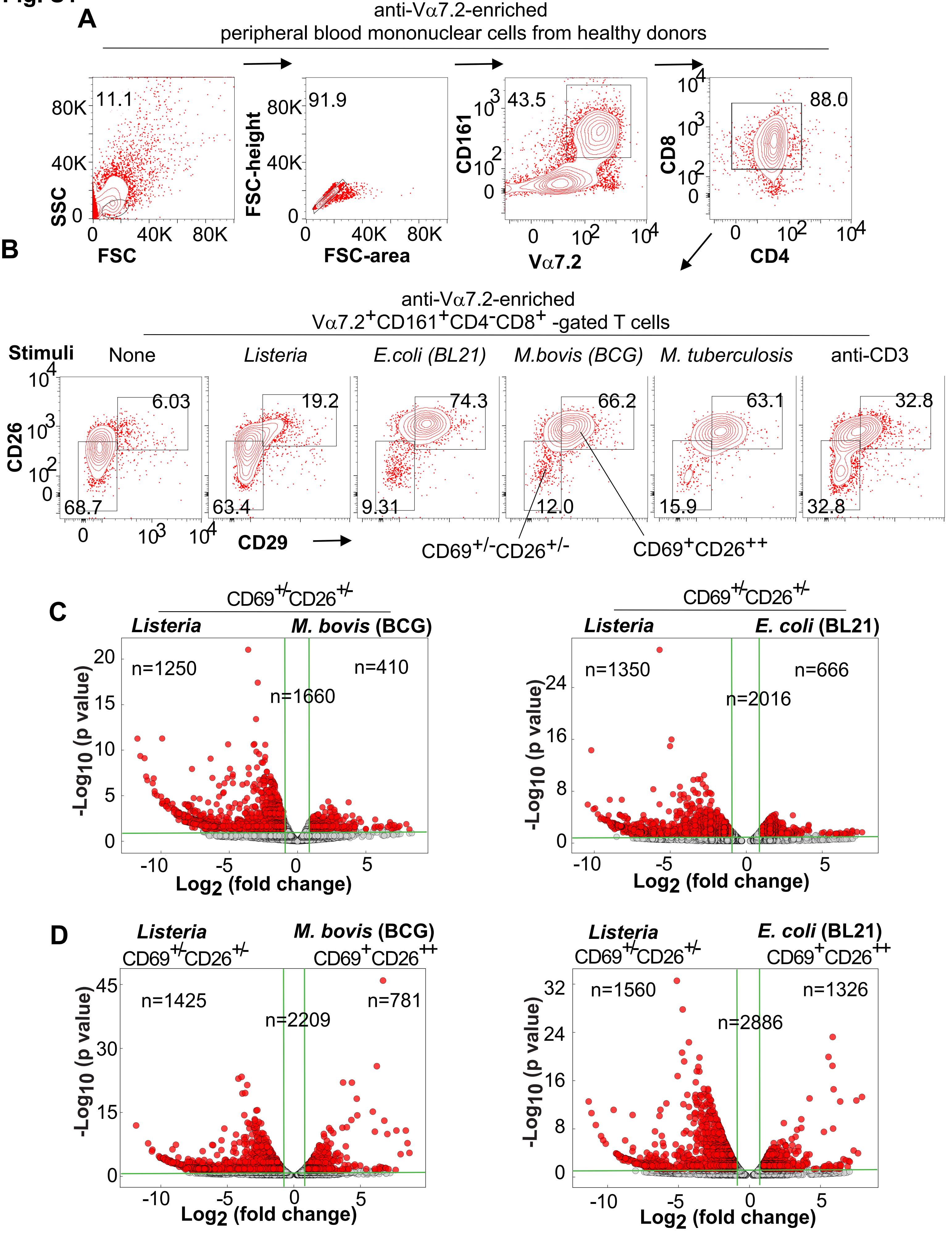
A
anti-Vα7.2-enriched
peripheral blood mononuclear cells from healthy donors
11.1
SSC
FSC
91.9
FSC-height
FSC-area
43.5
CD161
Vα7.2
88.0
CD8
CD4
B
anti-Vα7.2-enriched
Vα7.2+CD161+CD4-CD8+ -gated T cells
Stimuli
None
Listeria
E.coli (BL21)
M.bovis (BCG)
M. tuberculosis
anti-CD3
6.03
68.7
19.2
63.4
74.3
9.31
66.2
12.0
63.1
15.9
32.8
32.8
CD26
CD29
CD69+/-CD26+/-
CD69+CD26++
C
CD69+/-CD26+/-
Listeria
M. bovis (BCG)
n=1250
n=1660
n=410
-Log10 (p value)
Log2 (fold change)
CD69+/-CD26+/-
Listeria
E. coli (BL21)
n=1350
n=2016
n=666
-Log10 (p value)
Log2 (fold change)
D
Listeria
CD69+/-CD26+/-
M. bovis (BCG)
CD69+CD26++
n=1425
n=2209
n=781
-Log10 (p value)
Log2 (fold change)
Listeria
CD69+/-CD26+/-
E. coli (BL21)
CD69+CD26++
n=1560
n=2886
n=1326
-Log10 (p value)
Log2 (fold change)

Fig. S1

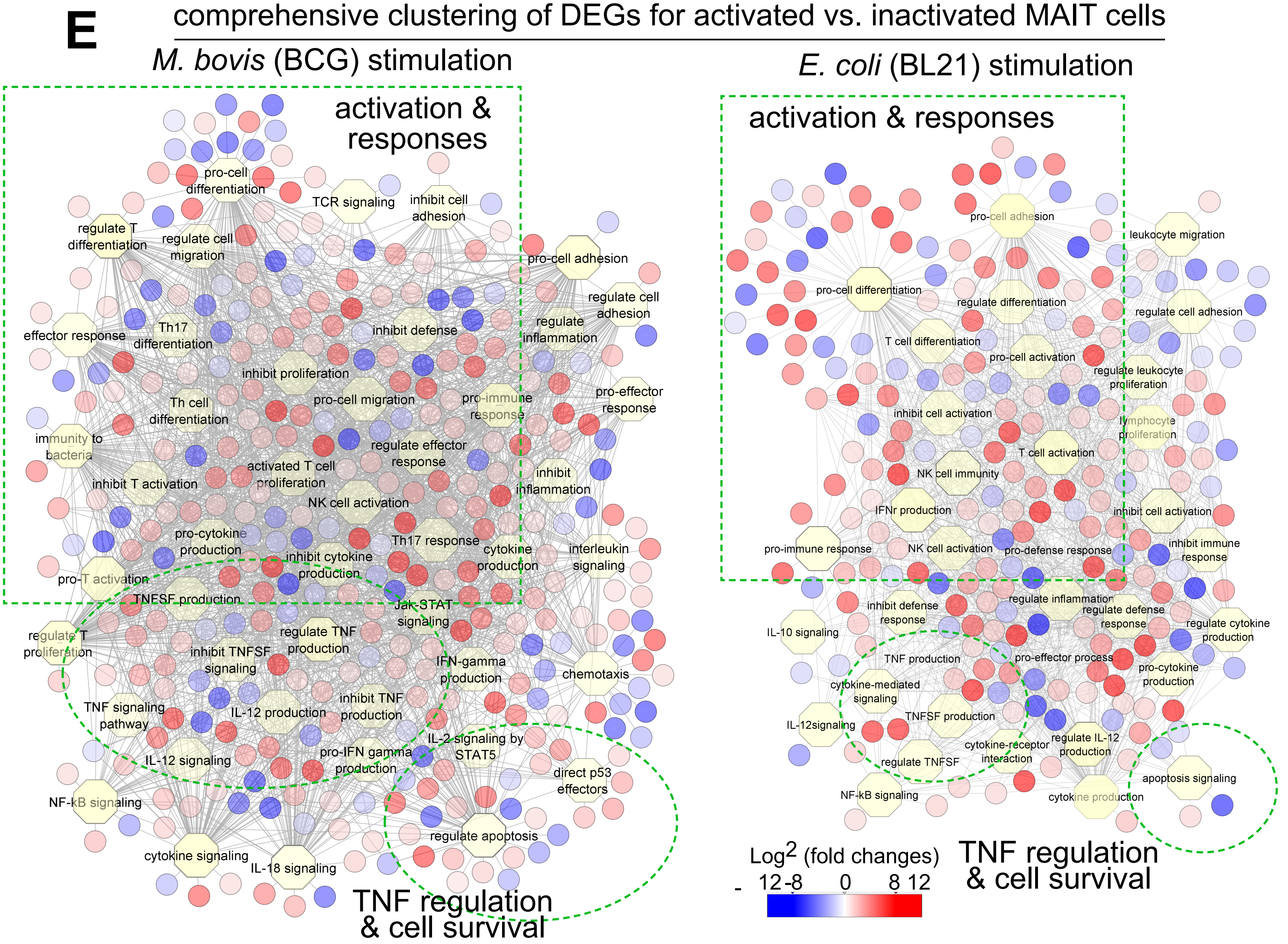
E
comprehensive clustering of DEGs for activated vs. inactivated MAIT cells
M. bovis (BCG) stimulation
activation & responses
pro-cell differentiation
TCR signaling
inhibit cell adhesion
regulate T differentiation
regulate cell migration
pro-cell adhesion
regulate cell adhesion
effector response
Th17 differentiation
inhibit defense
regulate inflammation
inhibit proliferation
pro-effector response
Th cell differentiation
pro-cell migration
pro-immune response
immunity to bacteria
regulate effector response
inhibit T activation
activated T cell proliferation
inhibit inflammation
NK cell activation
pro-cytokine production
Th17 response
cytokine production
interleukin signaling
inhibit cytokine production
pro-T activation
TNFSF production
Jak-STAT signaling
regulate T proliferation
regulate TNF production
inhibit TNFSF signaling
IFN-gamma production
chemotaxis
TNF signaling pathway
IL-12 production
inhibit TNF production
IL-2 signaling by STAT5
IL-12 signaling
pro-IFN gamma production
direct p53 effectors
NF-kB signaling
regulate apoptosis
cytokine signaling
IL-18 signaling
TNF regulation & cell survival
E. coli (BL21) stimulation
activation & responses
pro-cell adhesion
leukocyte migration
pro-cell differentiation
regulate differentiation
regulate cell adhesion
T cell differentiation
pro-cell activation
regulate leukocyte proliferation
inhibit cell activation
lymphocyte proliferation
T cell activation
NK cell immunity
IFNr production
inhibit cell activation
pro-immune response
NK cell activation
pro-defense response
inhibit immune response
inhibit defense response
regulate inflammation
regulate defense response
regulate cytokine production
IL-10 signaling
TNF production
pro-effector process
pro-cytokine production
cytokine-mediated signaling
TNFSF production
IL-12signaling
cytokine-receptor interaction
regulate IL-12 production
regulate TNFSF
apoptosis signaling
NF-kB signaling
cytokine production
Log2 (fold changes)
- 12 -8 0 8 12
TNF regulation & cell survival

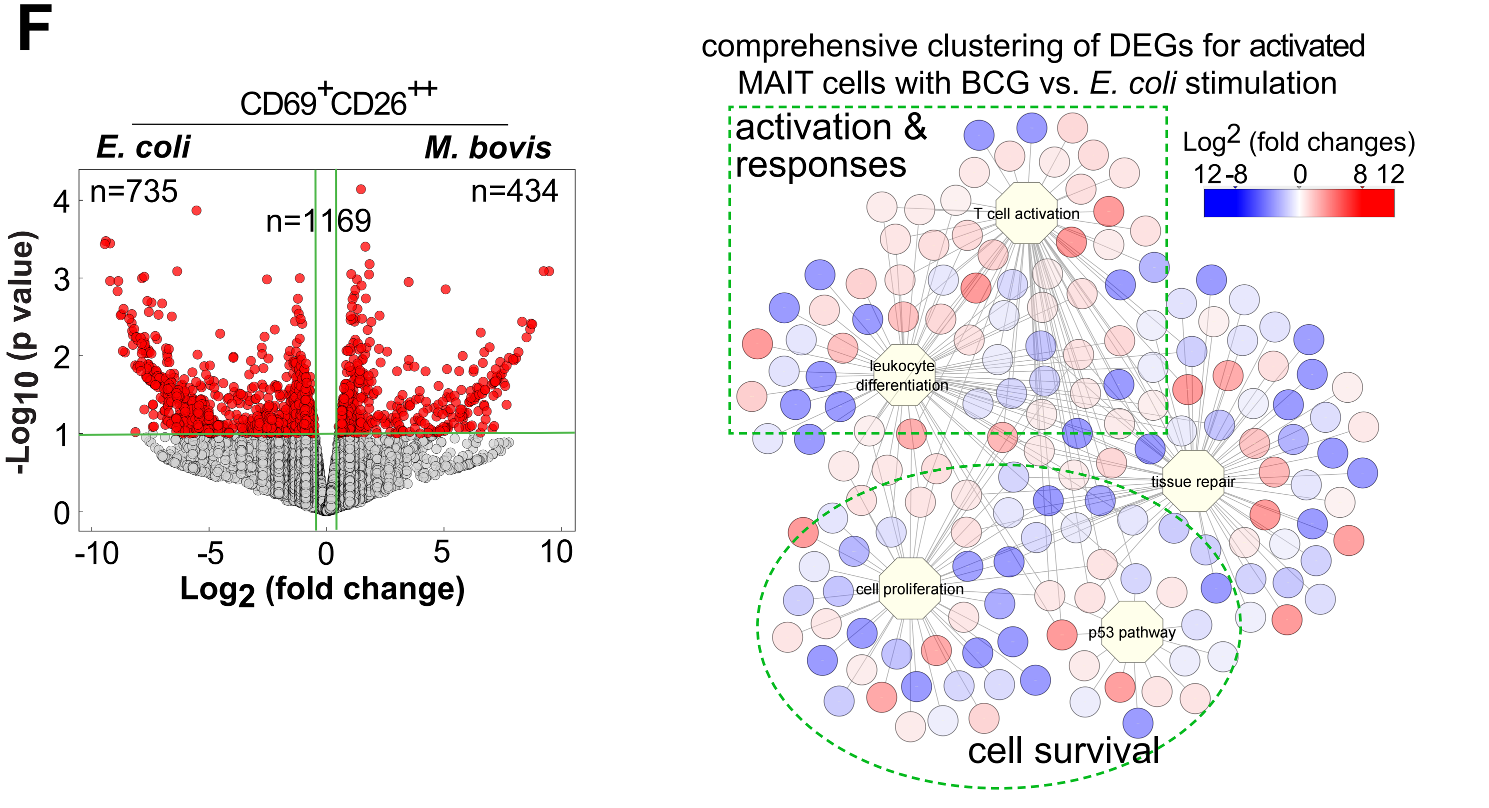
F
CD69+CD26++
E. coli
M. bovis
n=735
n=1169
n=434
-Log10 (p value)
4
3
2
1
0
-10
-5
0
5
10
Log2 (fold change)
comprehensive clustering of DEGs for activated MAIT cells with BCG vs. E. coli stimulation
activation & responses
Log2 (fold changes)
12 -8 0 8 12
T cell activation
leukocyte differentiation
tissue repair
cell proliferation
p53 pathway
cell survival

Fig. S2

A Gene pathways of cell proliferation and apoptosis highlighted with defferentially expressed genes in activated vs. inactivated MAIT cells upon *E. coli* (BL21) stimulation

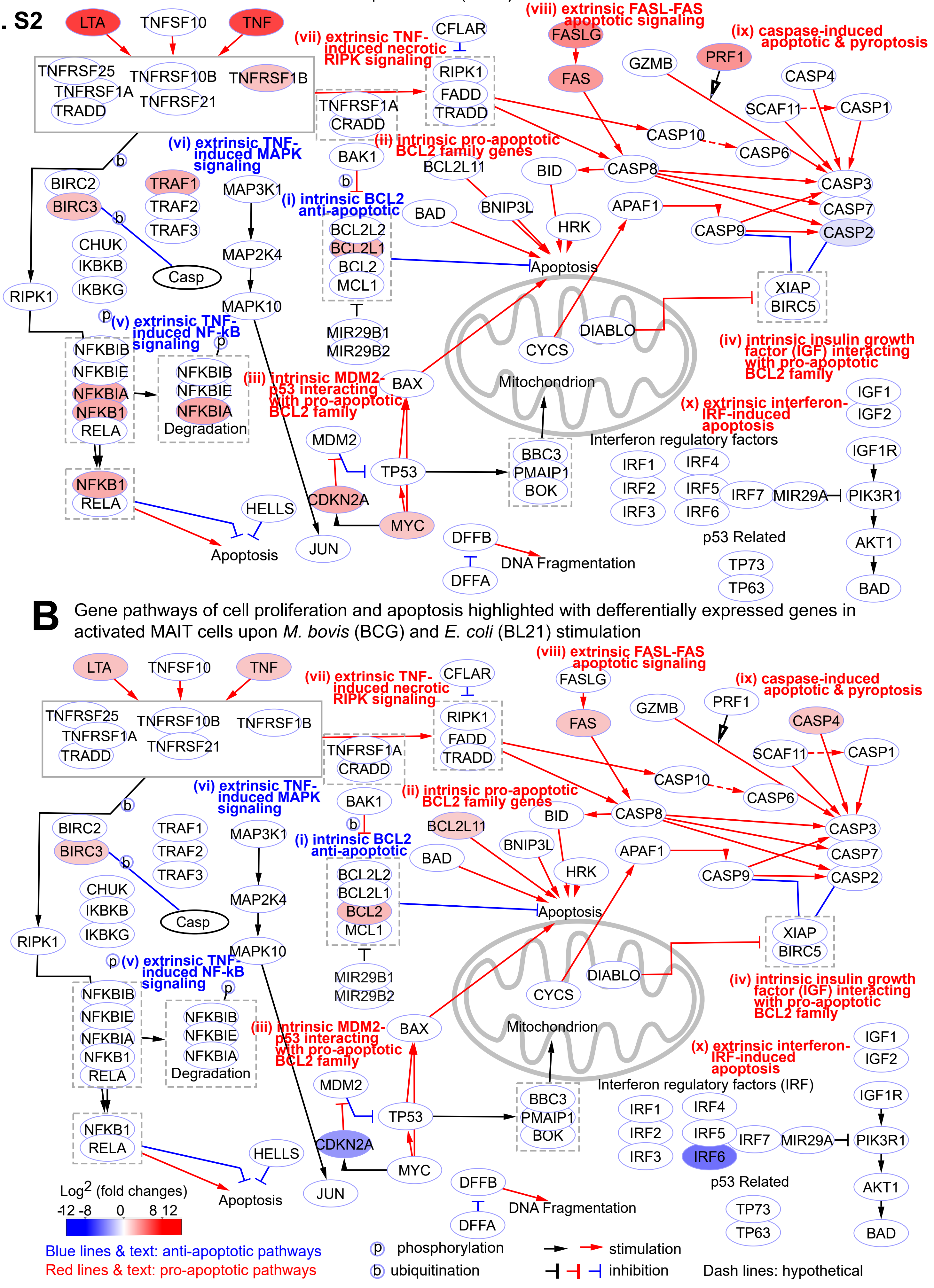

Fig. S3

A Gene pathways in cytotoxic T cells resonses highlighted with defferentially expressed genes in activated vs. inactivated MAIT cells upon *E. coli* (BL21) stimulation

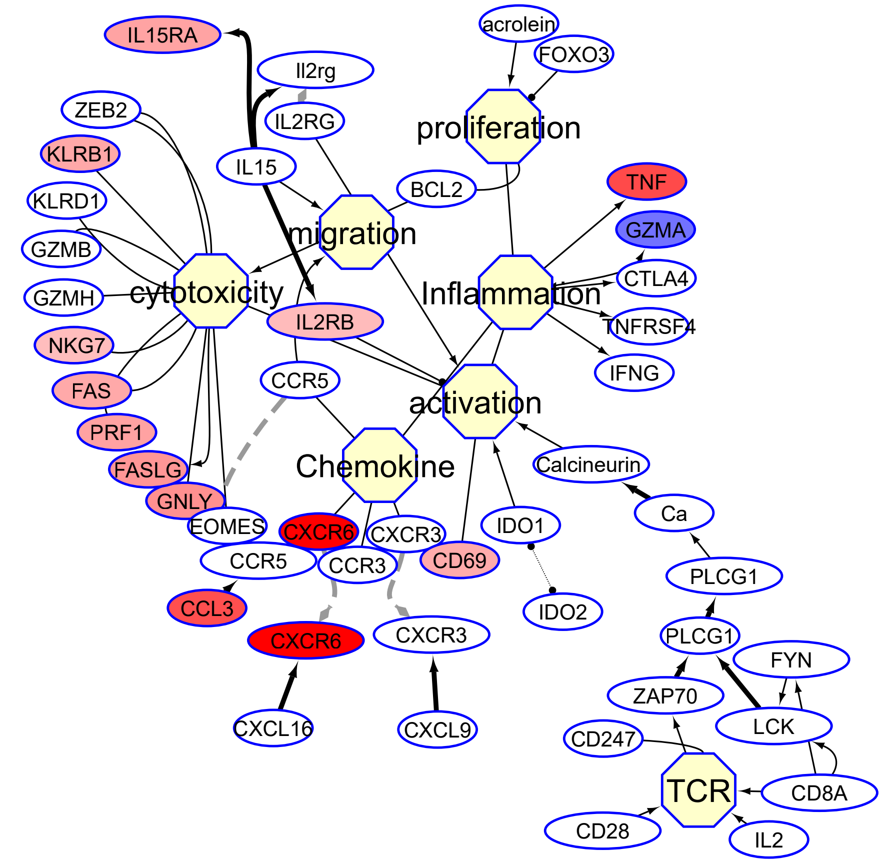

B Gene pathways in cytotoxic T cells resonses highlighted with defferentially expressed genes in activated MAIT cells upon *M. bovis* (BCG) and *E. coli* (BL21) stimulation

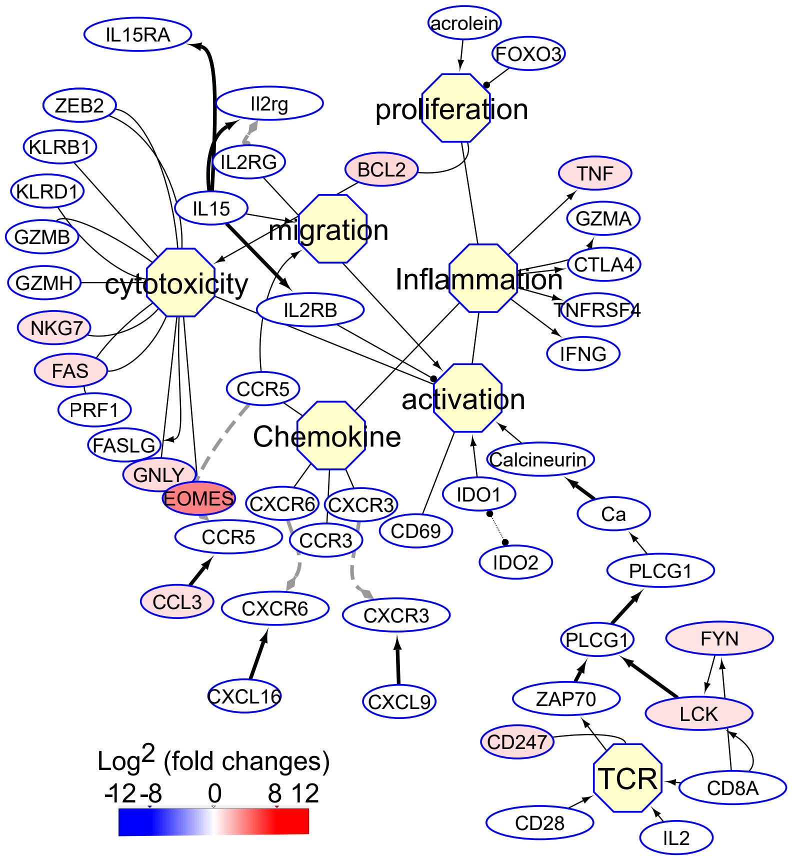

Supplement: Supplementary Figure 1 — Gating of activated versus inactivated CD8+ MAIT cells for RNA-seq analyses. CD8+ MAIT cells were gated on Vα7.2+CD161+CD4-CD8+ as Vα7.2+CD161+ gating has been used in multiple studies to detect MAIT cells (13, 29-32), especially the bacterial-activated MAIT cells (13, 33, 34) (A). % CD69+CD26++ CD8+ MAIT cells and CD69+/-CD26+/- cells are annotated, showing the cell populations sorted for RNA-sequencing and the strategy for gating activated MAIT cells (B). To determine whether the CD69+/-CD26+/- MAIT subsets at different bacterial incubation conditions show similar background response, we determined DEGs of CD69+/-CD26+/- inactivated MAIT cells between Listeria and BCG, or between Listeria and E. coli, suggesting high heterogeneity (C). High numbers of DEGs also occur with the activated MAIT subset (CD69+CD26++) upon BCG or E. coli incubations and inactivated MAIT subset (CD69+/-CD26+/-) with Listeria incubation (D). DEGs from the activated vs. inactivated MAIT cells responding to identical bacterial stimulations, the BCG and E. coli stimulation, were more clustered, respectively, to show genes associated with MAIT cell activation and survival (E). DEGs of activated MAIT cells from the direct comparison upon BCG vs. E. coli stimulation are expected to have a fewer number of genes and show more narrow clusters associated with MAIT cell reactivities (F). [file DataSheet_1.pdf]
